# pH-Dependent Surface Charge Modulation of Peptide-Coated Poly(lactic-co-glycolic Acid) (PLGA) Nanoparticle for Drug Delivery in Ovarian Cancer

**DOI:** 10.3390/molecules31172953

**Published:** 2026-08-23

**Authors:** Sylwia A. Dragulska, Mina Poursharifi, Benjamin Lesea-Pringle, Maxier Acosta Santiago, Caleb Mayes, Ying Chen, Maria Padron-Rhenals, Sandra Catalina Camacho, Kelsey Engelman, Olga Camacho-Vanegas, John A. Martignetti, Aneta J. Mieszawska

**Affiliations:** 1 Department of Chemistry and Biochemistry, Brooklyn College, The City University of New York, 2900 Bedford Avenue, Brooklyn, NY 11210, USAmaxier.santiago@thermofisher.com (M.A.S.); calebmayes@oakland.edu (C.M.); 2 Department of Genetics and Genomic Sciences, Icahn School of Medicine at Mount Sinai, 1425 Madison Avenue, New York, NY 10029, USA; zchenying@hotmail.com (Y.C.);; 3 Women’s Health Research Institute, Icahn School of Medicine at Mount Sinai, 1 Gustave L. Levy Pl., New York, NY 10029, USA; 4 Rudy Ruggles Research Institute, Western Connecticut Health Network, 131 West St., Danbury, CT 06810, USA

**Keywords:** pH-sensitive, nanoparticles, PLGA, ovarian cancer, peptide coating, EPR

## Abstract

The development of nanoparticle (NP)-based drug delivery systems that combine passive tumor targeting, physiological stability, and therapeutic efficacy remains a key challenge in cancer nanomedicine. Here, we report a pH-responsive peptide-functionalized poly(lactic-co-glycolic acid) (PLGA) NP system designed for cancer targeting. The PLGA core is coated with a short glutamic acid–lysine–histidine–phenylalanine x3 (EKHFFF) peptide shell, enabling tunable surface charge modulation around its isoelectric point and promoting environmental responsiveness. Physicochemical characterization confirms spherical NPs (~70–75 nm) with good colloidal stability, serum compatibility, and ion-dependent stability in physiological conditions. The peptide coating also provides pH-dependent modulation of the zeta potential. Evaluation of the NPs in ovarian cancer (OvCA) models, including immortalized and patient-derived cell lines (PDCLs), demonstrates efficient uptake across OvCA cell lines, with significantly enhanced internalization in PDCLs compared to immortalized cells. The EKHFFF nanoparticle (EKHFFF NP) induced minimal reactive oxygen species and nitric oxide production in macrophages, indicating low immunogenicity and favorable biocompatibility. Upon platinum loading (EKHFFF-Pt NP), the system exhibits potent cytotoxicity in both platinum-sensitive and platinum-resistant OvCA cell lines, outperforming carboplatin and showing comparable or improved efficacy relative to cisplatin in several cell lines. In vivo studies further demonstrate preferential tumor accumulation, sustained intratumoral retention, and measurable systemic circulation with a half-life of approximately 35 min.

## 1. Introduction

The functional versatility of short peptides has made them attractive components of cancer-directed therapeutic and delivery systems, where their sequence-dependent interactions can be exploited to modulate tumor cell responses and drug localization [1,2,3]. This specificity has enabled their application across diverse areas, including medicine [4,5,6], skincare [7], and biotechnology [8]. Despite inherent limitations such as short half-life and limited stability under physiological conditions, peptide engineering strategies have been developed to enhance bioavailability, stability, and overall drug-like properties [9,10,11].

Peptides contribute to cancer therapy through multiple mechanisms, including inhibition of tumor growth and metastasis [12]. Specifically, peptides can be designed to interfere with proteins involved in oncogenic signaling pathways, thereby suppressing tumor progression and metastatic spread. Conjugation of peptides with fluorescent or radiolabeled probes has also enabled their use as molecular imaging agents for tumor localization and treatment monitoring [13]. Peptides that bind to cancer-specific biomarkers with high affinity can serve as molecular imaging probes, improving the sensitivity and specificity of cancer detection [14]. Furthermore, select peptides exhibit immunomodulatory activity, supporting their use in cancer immunotherapy by enhancing immune recognition and elimination of cancer cells [15,16]. Peptides also serve as versatile building blocks for the construction of peptide-based nanomaterials with tunable size, shape, and surface properties [17,18,19]. Such nanomaterials exhibit high surface area-to-volume ratios and have been explored for applications in drug delivery, biosensing, tissue engineering, and imaging [20,21,22].

The expanding clinical presence of peptide- and oligonucleotide-based therapeutics is reflected in the 2020 FDA approvals, which included 6 such agents among 53 newly approved drugs. [23,24]. Among peptide-based strategies, pH-sensitive peptides have emerged as particularly attractive candidates for targeting acidic pathological tissues [25,26,27,28,29]. Tissue acidification is a hallmark of several disease states, including cancer, inflammation, infection, and arthritis [30,31,32]. In tumors, extracellular acidification increases during disease progression as cancer cells shift toward glycolytic metabolism and upregulate carbonic anhydrase activity, processes that contribute to tumor growth and malignancy. Consequently, pH serves as a critical indicator and modulator of disease progression [33,34,35].

The acidic tumor microenvironment has motivated the development of pH-responsive drugs, prodrugs, and delivery systems [36,37]. Prior studies have demonstrated the utility of acid-labile linkages between therapeutic agents and their carriers, as well as pH-responsive functional groups that enable controlled drug activation or release [38,39,40,41].

pH-responsive nanoparticles (NPs) are promising systems for targeted cancer therapy because they exploit the acidic tumor microenvironment to trigger selective drug release. Major classes include polymeric NPs [42,43], liposomes [44], mesoporous silica NPs [45], dendrimers [46], metal–organic frameworks [47], and inorganic NPs such as calcium phosphate [48] or zinc oxide [49]. These nanocarriers are typically engineered with acid-sensitive bonds or protonatable groups that enable cargo release under acidic conditions found in tumors or intracellular endosomes. By improving site-specific delivery and minimizing off-target toxicity, pH-responsive NPs can enhance the therapeutic efficacy and safety of anticancer treatments. However, one of the main remaining challenges is minimizing the toxicity of the NP drug carrier, highlighting the need for continued development and rigorous evaluation to facilitate clinical translation [50].

Herein, we present a polymeric NP stabilized with a pH-responsive short peptide. The core of the NP consists of poly(lactic-co-glycolic acid) (PLGA) polymer, which is widely used for NP synthesis due to its excellent biocompatibility features and tunable drug release kinetics [51]. The EKHFFF peptide shell contains three pH-responsive amino acids—glutamic acid (E), lysine (K), and histidine (H)—while the aromatic phenylalanine (F) residues promote peptide self-assembly on the PLGA surface through hydrophobic and π-π interactions. The EKHFFF NP is completely biodegradable, and its surface charge changes in response to physiological and tumoral microenvironments. As a proof of concept, the efficacy of the NP was evaluated by encapsulating Pt(II) therapy and formulating EKHFFF-Pt NPs.

## 2. Results

### 2.1. EKHFFF NP Characterization

#### 2.1.1. Size and Morphology

**Figure 1:**
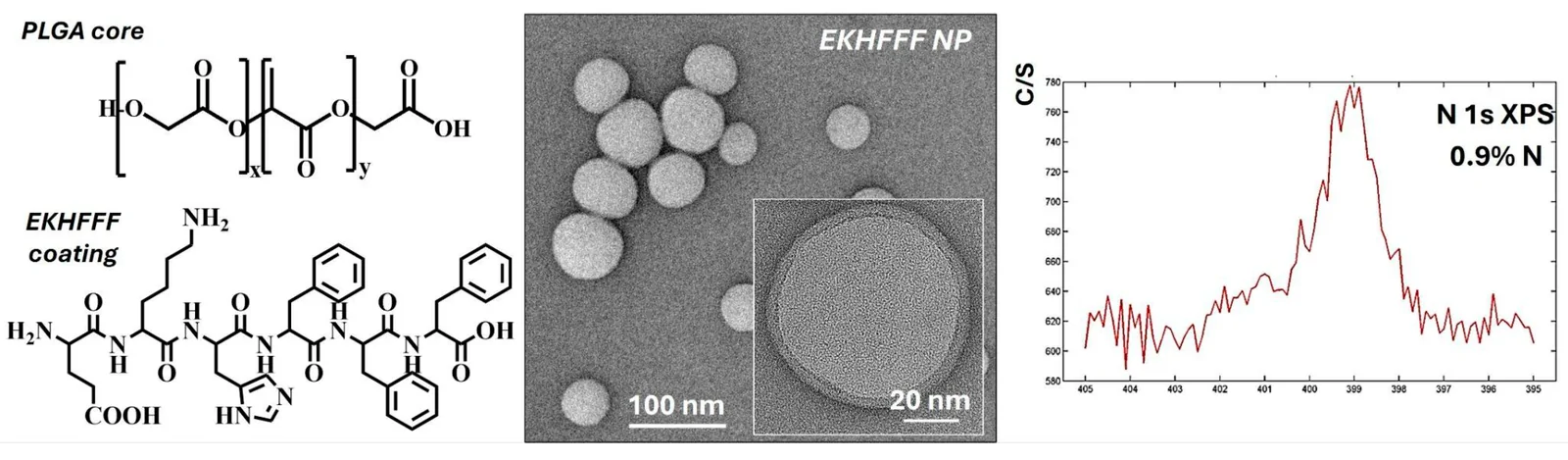
PLGA polymer (**left top**), and EKHFFF peptide (**left bottom**). TEM image (**middle**) and XPS spectrum (**right**) of EKHFFF NP surface.

EKHFFF NPs were synthesized using a well-established nanoprecipitation method, a widely used and reproducible bottom-up technique for preparing polymeric nanocarriers [52]. Briefly, PLGA dissolved in acetonitrile was added dropwise to an aqueous solution of EKHFFF peptide (Figures S1 and S2) at a polymer-to-peptide molar ratio of 1:1.7 and a temperature of 37 °C. The mixture was then stirred overnight at room temperature, during which the organic solvent evaporated and the EKHFFF NPs spontaneously self-assembled (Figure 1).

EKHFFF NP size and morphology were characterized by transmission electron microscopy (TEM). As shown in Figure 1, the NPs are spherical and exhibit a uniform 2–3 nm shell of the EKHFFF peptide coating on the surface of the PLGA core. Our previous studies have reported that this shell is observed only in peptide-coated NPs and is absent in plain PLGA NPs [53]. The average diameter of the EKHFFF NPs measured by TEM was 74 ± 2.7 nm. EKHFFF NPs exhibit a size compatible with tumor accumulation mediated by the enhanced permeability and retention (EPR) effect. Nanoparticles within approximately 30–200 nm range are generally considered favorable for sustained intratumoral retention through the EPR effect as they are large enough to avoid rapid renal clearance while remaining capable of extravasation through leaky tumor vasculature [54]. Most solid tumors exhibit abnormal and poorly organized vasculature, resulting in increased permeability to nanoscale materials and thereby facilitating preferential NP accumulation within tumors [55,56].

#### 2.1.2. Elemental Composition of the NP’s Coating

In addition to TEM imaging, the presence of peptides on the surface of the NPs was qualitatively confirmed by X-ray photoelectron spectroscopy (XPS). The penetration depth of XPS is limited to a few nanometers, and the only nitrogen-containing component of the NPs is the peptide. The nitrogen spectrum shown in Figure 1 confirms the presence of a peptide as a shell on the NP surface. The control spectrum of the Si wafer (Figure S3, top), together with the curve fitting of the carbon (Figure S3, middle) and nitrogen (Figure S3, bottom) spectra, is presented in the Supplementary Materials.

For quantitative analysis of the shell/core molar ratio, elemental analysis was performed on both EKHFFF coated and uncoated NPs. Based on these data, the molar ratio of peptide shell to PLGA core was calculated to be 1:4.4. The corresponding elemental analysis results are presented in Figure S4.

#### 2.1.3. Surface Charge

**Figure 2:**
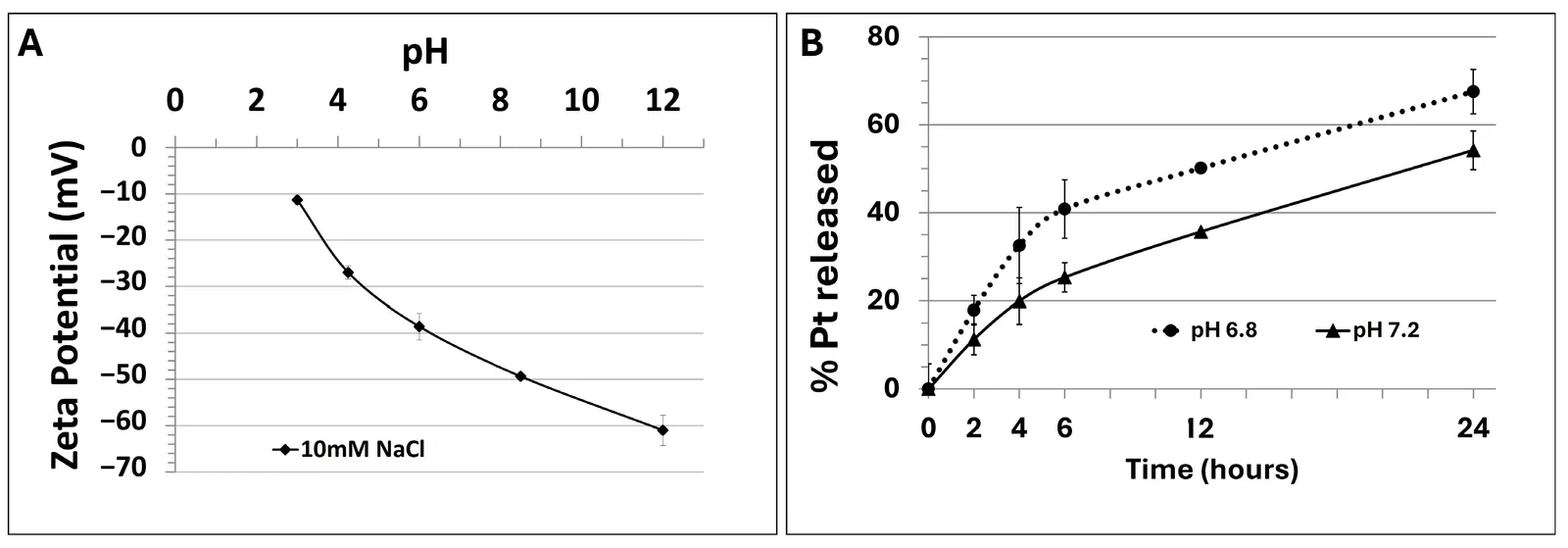
(**A**) Zeta potential of EKHFFF NPs measured in 10 mM NaCl solution at different pH values. (**B**) pH-dependent Pt(II) release profile of EKHFFF-Pt nanoparticles, showing cumulative Pt(II) release at pH 6.8 and pH 7.2 over 24 h at 37 °C. Data are presented as mean ± SD from three independent experiments (*n* = 3).

The surface charge of EKHFFF NPs was evaluated under varying pH conditions to assess the pH responsiveness of the peptide coating. Figure 2A demonstrates that the nanoparticles exhibited a negative zeta potential across the tested pH range (3–12), with values becoming progressively more negative from −11 ± 0.98 mV at pH 3 to −61 ± 3.27 mV at pH 12. The pH-dependent changes in zeta potential arise from protonation and deprotonation of the α-amino, α-carboxyl, and ionizable side-chain functional groups of the EKHFFF peptide (Table 1). Importantly, the calculated isoelectric point (pI = 6.96) and the corresponding net charge describe the isolated EKHFFF peptide rather than the peptide-coated nanoparticles. Under acidic conditions, the free EKHFFF peptide carries a net positive charge, which gradually decreases as the pH increases due to progressive deprotonation. In contrast, the experimentally measured zeta potential reflects the electrokinetic potential of the entire nanoparticle, which is determined not only by the peptide coating but also by the PLGA core, hydration layer, adsorbed ions, and the electrical double layer. Consequently, the nanoparticles remain negatively charged throughout the investigated pH range because of the contribution of ionized PLGA carboxyl groups. Nevertheless, protonation of the peptide under acidic conditions markedly attenuates the negative surface charge, resulting in a progressive shift toward less negative zeta potential values. These findings demonstrate pH-dependent modulation of nanoparticle surface properties and confirm that the EKHFFF coating retains its pH-responsive behavior after conjugation to the nanoparticle surface.

#### 2.1.4. Release Profile of Pt(II) from EKHFFF-Pt NP

The EKHFFF-Pt NP was formulated using a PLGA-Pt hybrid, which was synthesized according to our previously reported method [57,58]. To determine whether the pH-responsive surface properties of the EKHFFF coating influence drug release, Pt(II) release was evaluated under physiological and mildly acidic conditions (Figure 2B). Briefly, EKHFFF-Pt NPs were suspended in buffer solutions at the indicated pH values and incubated at 37 °C for 72 h, and the Pt(II) content was quantified by atomic absorption spectroscopy (AAS) to determine cumulative Pt(II) release. To emphasize the pH-dependent differences during the initial release phase, Figure 2B presents the first 24 h of the release study, while the complete 72 h release profile is shown in Figure S5. EKHFFF-Pt nanoparticles released Pt(II) more rapidly at pH 6.8 than at physiological pH 7.2 during the first 24 h of incubation. After 24 h, cumulative Pt(II) release reached approximately 68% at pH 6.8 compared with 55% at pH 7.2. Thereafter, Pt(II) release at pH 6.8 approached a plateau, whereas release at pH 7.2 continued to increase gradually throughout the remainder of the study, reaching approximately 76% after 72 h. These findings demonstrate that mildly acidic conditions promote earlier Pt(II) release from the nanoparticle formulation. Such pH-dependent release is advantageous for tumor-selective drug delivery, as the slightly acidic extracellular tumor microenvironment may facilitate earlier drug release at the tumor site while reducing premature drug leakage under physiological conditions.

### 2.2. Stability of the EKHFFF NP

#### 2.2.1. The Effect of Ionic Solutions

Next, as part of the evaluation of the NP for biological applications, the aggregation behavior of the EKHFFF NPs was investigated under varying cation valences and ionic strengths, reflecting physiologically relevant conditions found in human blood. The hydrodynamic size and turbidity of the NPs were measured in the presence of monovalent (Na^+^) and divalent (Ca^2+^) cations, which are among the most abundant physiologically relevant cations. Briefly, to monitor the physicochemical stability of EKHFFF NPs, a 1.2 mg/mL NP solution was diluted in NaCl and CaCl_2_ solutions of different ionic strengths. The results were compared to uncoated PLGA NPs.

**Figure 3:**
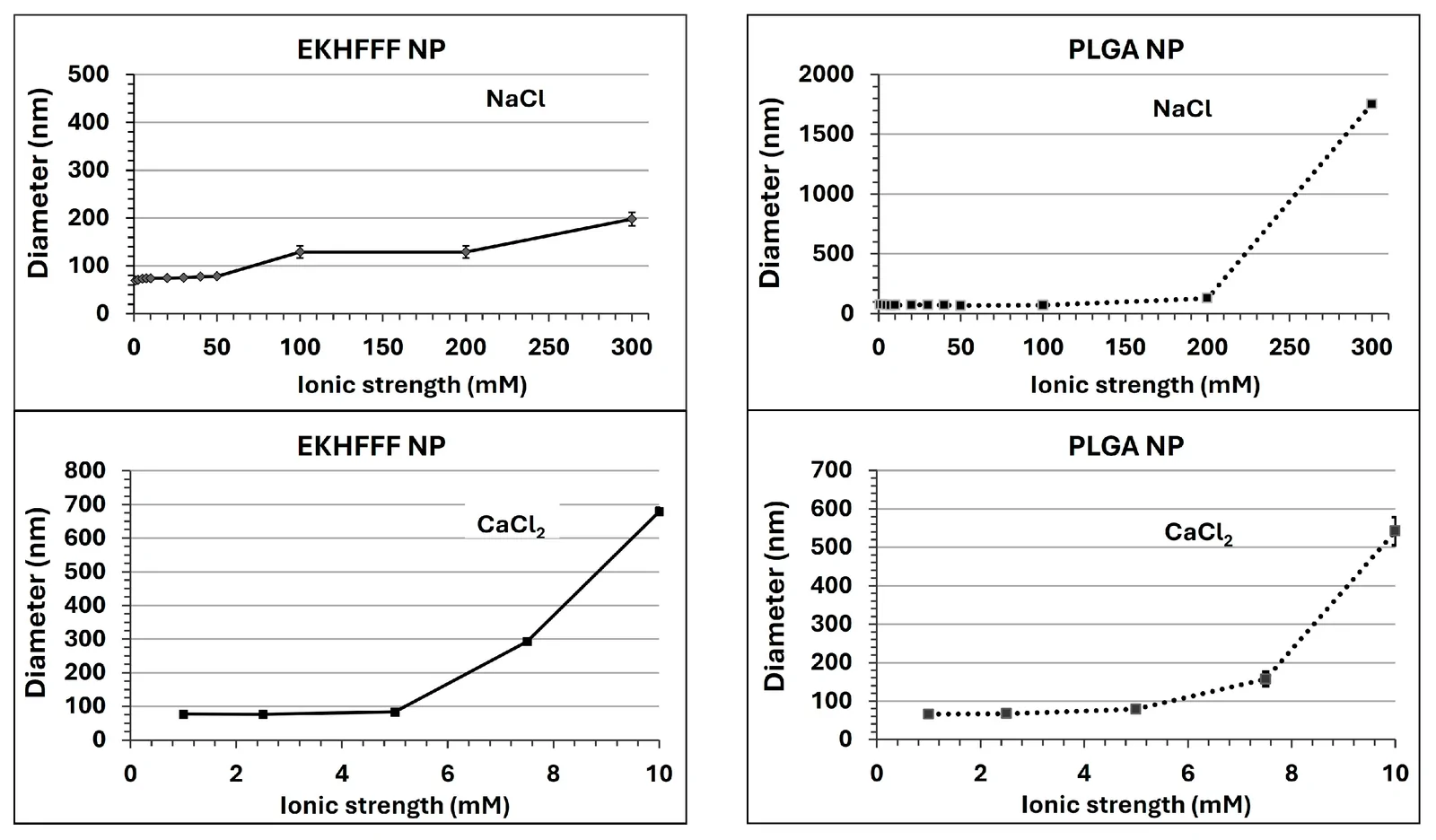
The EKHFFF NP and PLGA NP sizes under different ionic strengths of Na^+^ and Ca^2+^ ions at pH 7. Data are presented as the mean ± SD from three independent experiments (*n* = 3).

The ionic strength of NaCl solution was gradually increased to 300 mM. The EKHFFF NPs remained stable at concentrations between 30 and 200 mM, suggesting good stability of the NPs under physiological conditions, where Na^+^ concentration is ~150 mM. The average hydrodynamic diameter increased slightly to 200 nm at 300 mM NaCl (Figure 3, top left). Notably, the diameter of the PLGA NPs without any coating increased drastically to 1500 nm at 200 mM, indicating complete aggregation of the NPs (Figure 3, top right). Similarly, the EKHFFF NPs were tested under different ionic strengths of CaCl_2_ solutions. The extracellular concentration of the Ca^2+^ ion is approximately 1.2–1.3 mM and both, the EKHFFF NP and uncoated PLGA NP were stable and did not aggregate at this ion concentration. However, the aggregation of both NPs started at ~5 mM concentration of CaCl_2_, and increased from 72 nm to approximately 700 nm between 5 and 10 mM CaCl_2_ (Figure 3, bottom panel).

#### 2.2.2. Turbidity Measurements

**Figure 4:**
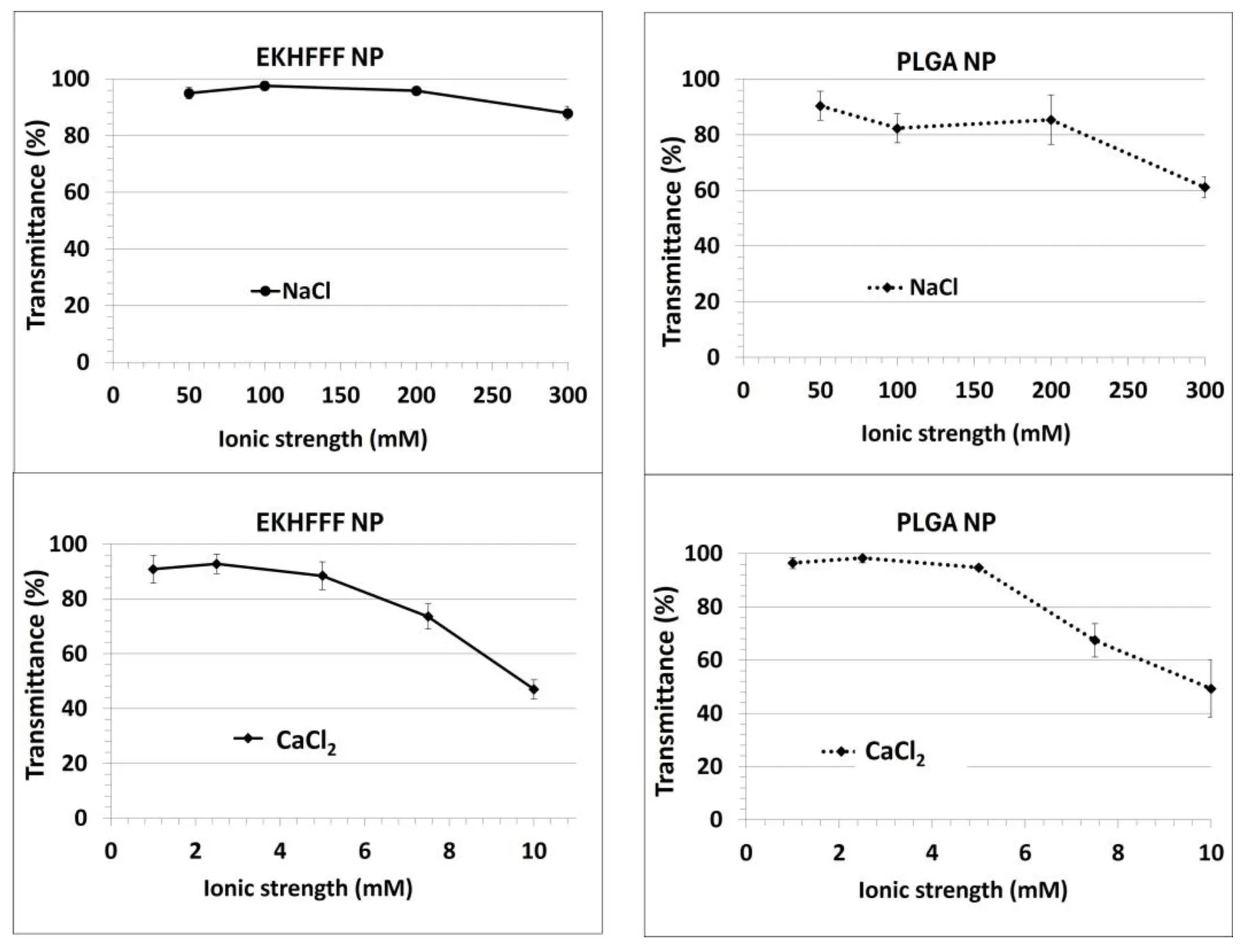
Turbidity analysis of (**left**) EKHFFF and (**right**) PLGA NPs under varying ionic conditions. Percent transmittance was measured as a function of ionic strength in NaCl (0–300 mM) and CaCl_2_ (1–10 mM) solutions to evaluate colloidal stability of the NPs. Error bars represent standard deviation from independent measurements (*n* = 3).

EKHFFF NP colloidal stability was also assessed by measuring the turbidity of NP suspensions in various ionic solutions over time (Figure 4, top panel). Transmittance of the NP in 50–300 mM NaCl solutions decreased progressively with increasing NaCl concentration. At 300 mM NaCl, EKHFFF NP suspension maintained relatively high transmittance (~90%), indicating limited aggregation. In contrast, PLGA NP suspensions exhibited substantially higher turbidity under the same conditions, with transmittance values of ~80% at 100–200 mM and decreasing to ~55% at 300 mM NaCl.

Turbidity was also evaluated in CaCl_2_ solutions (1–10 mM). Both EKHFFF and PLGA NP suspensions showed a marked decrease in transmittance beginning at ~5 mM Ca^2+^, reaching ~50% transmittance at 10 mM, indicating significant aggregation (Figure 4, bottom panel). These results demonstrate that the stabilizing effect of the EKHFFF peptide coating is more pronounced in the presence of monovalent ions such as Na^+^, a major component of blood plasma, where low aggregation is desirable for physiological applications. In contrast, divalent cation induced rapid destabilization, with aggregation occurring at much lower concentrations. The kinetics of transmittance as a function of time for both NaCl and CaCl_2_ are presented in the Supplementary Materials (Figure S6).

This behavior is consistent with ion-specific effects described in the literature, where ion valence and hydration influence the colloidal stability of macromolecular and nanoparticle systems. Divalent cations are generally more effective than monovalent ions at screening electrostatic repulsion and promoting interparticle interactions, which can lead to aggregation at lower concentrations [59,60]. Consistent with these observations, EKHFFF-coated nanoparticles remained colloidally stable over the investigated NaCl concentration range, whereas Ca^2+^ induced aggregation at substantially lower concentrations.

Overall, these findings highlight the critical role of the ionic environment in modulating NP stability. The EKHFFF NPs remain colloidally stable under physiologically relevant monovalent ion concentrations but become increasingly susceptible to aggregation in the presence of elevated ionic strength or higher-valence cation. Together with the pH-dependent surface charge modulation mediated by the ionizable glutamic acid and lysine residues of the EKHFFF peptide, ionic strength and cation valence are key environmental factors governing the surface properties and colloidal stability of EKHFFF NPs.

### 2.3. Biological Evaluation of the EKHFFF NP

#### 2.3.1. Confocal Visualization of EKHFFF NP Uptake by Immortalized OvCA Cells

**Figure 5:**
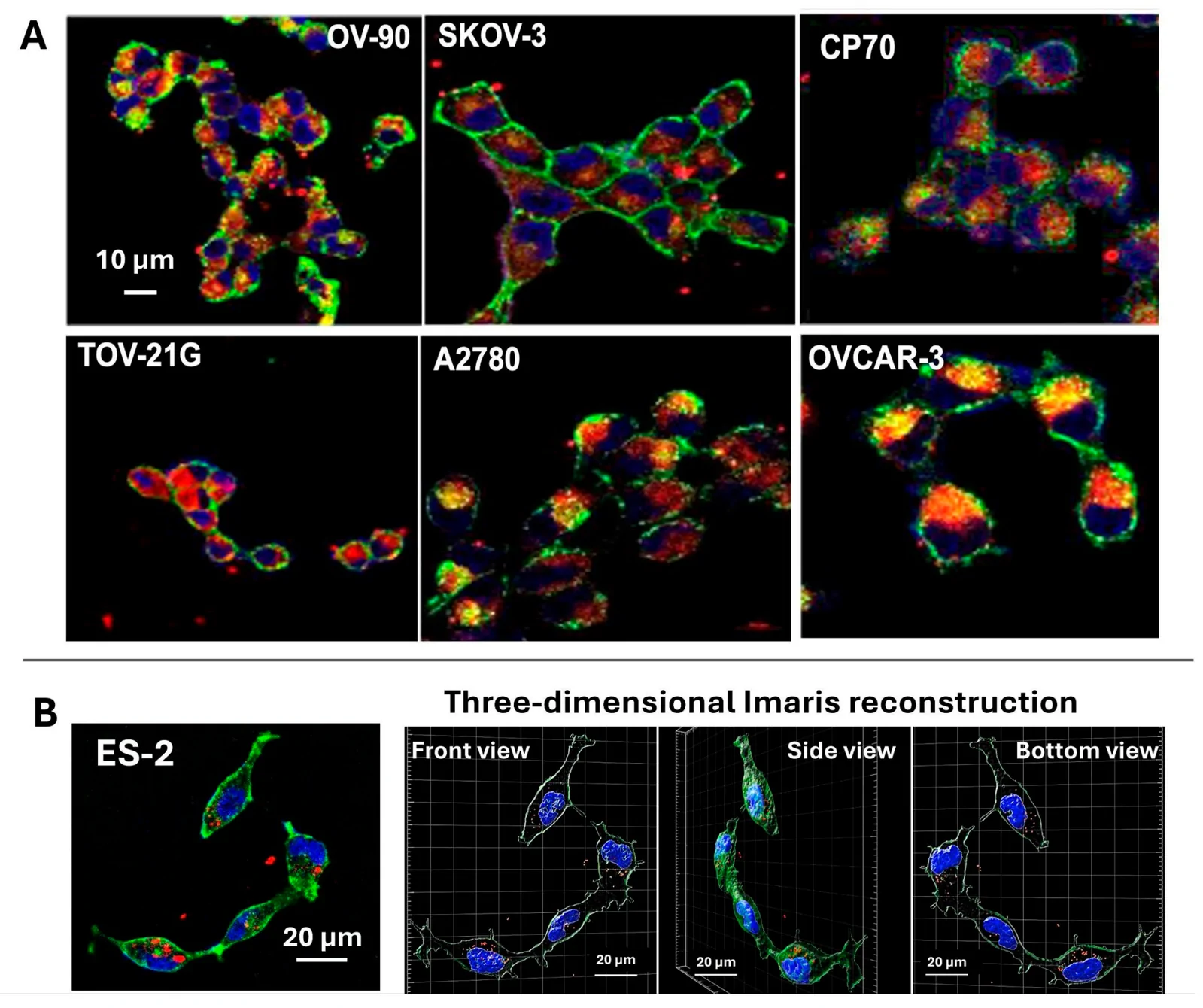
Confocal fluorescence imaging and three-dimensional Imaris reconstruction of EKHFFF-Cy5.5 nanoparticles in ovarian cancer cells. (**A**) Representative confocal microscopy images of ovarian cancer cell lines. EKHFFF-Cy5.5 nanoparticles are visualized in red, while cell membranes and nuclei are labeled with WGA–Alexa Fluor 488 (green) and DAPI (blue), respectively. (**B**) Representative ES-2 confocal image and corresponding three-dimensional Imaris reconstruction showing front, side, and bottom views. Scale bars: 10 µm (**A**) and 20 µm (**B**).

As an initial step toward biological evaluation, the cellular uptake and intracellular localization of fluorescently labeled EKHFFF-Cy5.5 NPs were examined in a panel of ovarian cancer cell lines by confocal laser scanning microscopy. The data presented in Figure 5 demonstrate efficient NP internalization across all tested cell lines, with the Cy5.5 fluorescence signal (red) distributed throughout the cytosol. Three-dimensional Imaris reconstruction (Figure 5B, and Movie S1 in the Supplementary Materials) of ES-2 cells further confirmed that the nanoparticles were localized within the cell volume rather than remaining associated with the cell surface, supporting efficient intracellular uptake.

#### 2.3.2. Flow Cytometric Analysis of EKHFFF NP Uptake by OvCA and PDCLs

In addition, we evaluated EKHFFF-Cy5.5 NP uptake in OvCA immortalized cells and PDCL using flow cytometry. Patient-derived OvCA cells were added as a key step toward bridging the gap between simplified in vitro models and clinically relevant tumor biology. Unlike immortalized cell lines, PDCLs preserve key tumor features, including genetic heterogeneity, variable receptor expression, and drug resistance mechanisms [61], all of which can influence nanoparticle internalization and intracellular trafficking. Assessing uptake in these models provides a more realistic measure of NP performance and enhances the predictive value of preclinical studies, supporting the translation of NP-based platforms to clinical applications.

**Figure 6:**
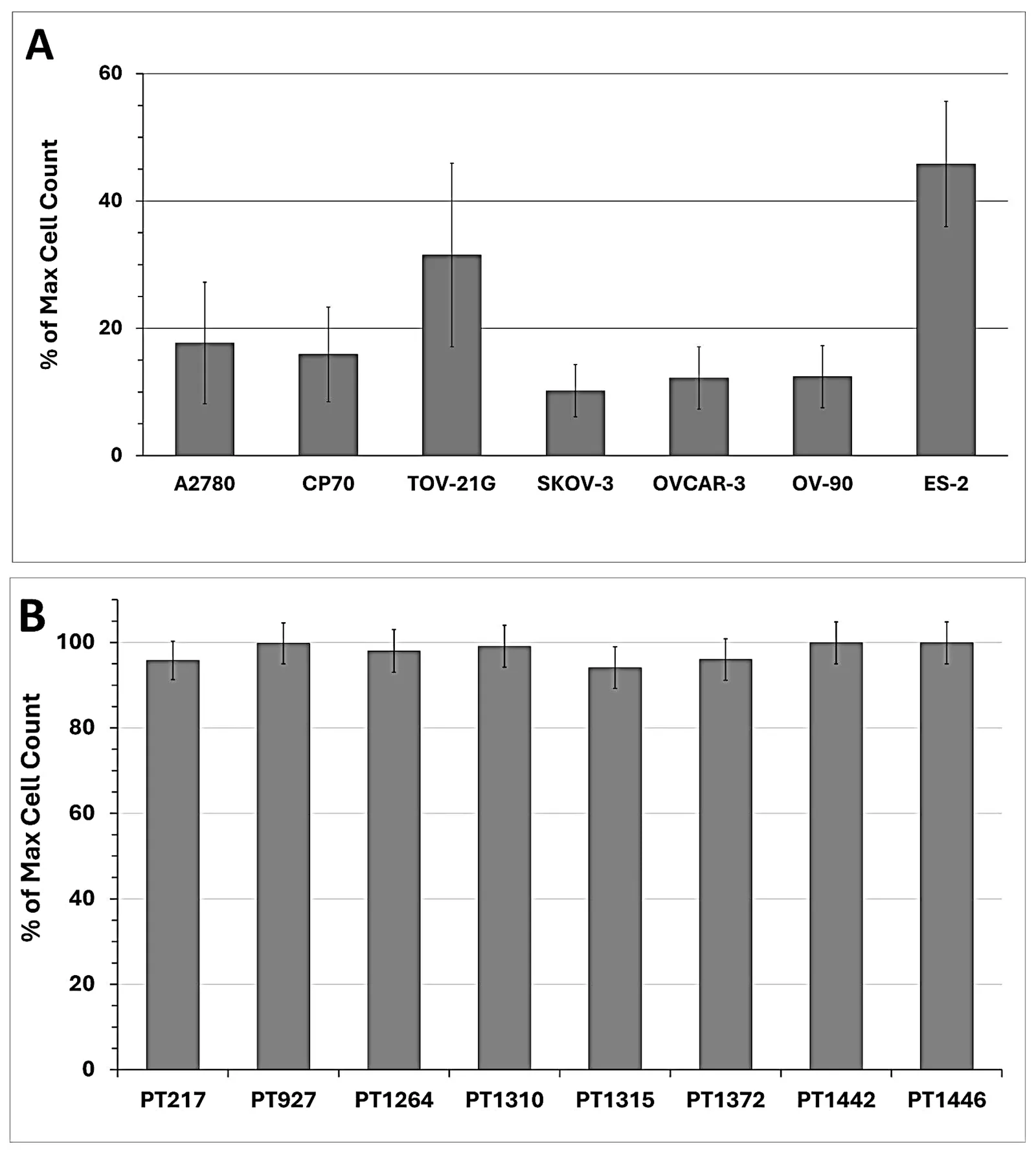
Quantification of EKHFFF-Cy5.5 NP uptake by flow cytometry (FACS). (**A**) NP uptake in OvCA immortalized cell lines; (**B**) NP uptake in patient-derived cell lines (PDCLs). Cells were incubated with the NPs for 15 min under standard culture conditions, followed by washing to remove unbound nanoparticles prior to analysis. Histograms and/or mean fluorescence intensity (MFI) values demonstrate enhanced nanoparticle internalization in PDCLs compared to immortalized cell lines. Data are presented as the mean ± SD from independent experiments (*n* = 3).

Among the immortalized cell lines (Figure 6A), ES-2 and TOV-21G showed the highest percent positivity for EKHFFF NP, 38.8% and 21.3%, respectively, while CP70, OV-90, OVCAR-3, SKOV-3 and A2780 exhibited significantly lower percent positivity (10.6%, 8.95%, 8.75%, 7.3%, and 10.9%, respectively), showing that internalization is cell-dependent. The uptake of EKHFFF-Cy5.5 NP was evaluated in eight different OvCA PDCLs: PT217, PT927, PT1264, PT1310 (serous ovarian cancer), PT1315 (serous endometrial cancer), PT1372, PT1442, and PT1446. The results are presented in Figure 6B and show more than 94% positivity for all eight PDCLs, which is much higher than in immortalized OvCA cells. The corresponding numerical data are provided in Figure S7 of the Supplementary Materials.

The higher NP uptake observed in PDCLs compared with immortalized cell lines may be explained by several factors. First, patient-derived cells often preserve heterogeneous and patient-specific expression of cell surface receptors involved in uptake and signaling, including integrins, growth factor receptors, and scavenger receptor pathways [62], so increased or heterogeneous expression may enhance binding of the NP and internalization. Second, they typically exhibit more active and diverse endocytic pathways [63]. Immortalized cell lines adapt to long-term culture and can downregulate or homogenize uptake mechanisms, whereas patient-derived cells preserve tumor-specific uptake behaviors, including macropinocytosis and receptor-mediated endocytosis, both of which may contribute to increased NP internalization. Third, membrane composition and fluidity differ. Patient-derived cells often have altered lipid composition and membrane organization associated with malignancy [64], which can facilitate NP adhesion and entry. Fourth, these cells better preserve clinically relevant tumor metabolic and stress states, including altered nutrient utilization and uptake programs. Tumor cells frequently upregulate endocytic and membrane-trafficking pathways to support growth and meet increased metabolic demands [63,65], which may also contribute to enhanced nanoparticle uptake. Finally, patient-derived cells maintain intratumoral heterogeneity [61], meaning subpopulations with particularly high uptake can exist, raising the total measured signal. In contrast, immortalized lines are more uniform and may underestimate uptake seen in real tumors. Overall, higher uptake in patient-derived cells suggests that the EKHFFF NPs interact effectively with tumor cells that more closely reflect the clinical setting. This observation supports further investigation of their translational potential.

#### 2.3.3. Toxicity of the EKHFFF NP

Next, we measured reactive oxygen species (ROS) and nitric oxide (NO) production in RAW 264.7 macrophages to assess whether EKHFFF NPs induce an inflammatory or immunostimulatory response. RAW 264.7 cells are murine macrophage-like cells that respond strongly to foreign materials [66]. Upon activation (e.g., by LPS or reactive nanomaterials), they produce nitric oxide via inducible nitric oxide synthase (iNOS). NO is a key signaling and effector molecule in inflammation, and its levels therefore serve as a sensitive readout of macrophage activation.

In addition to NO, ROS are a central component of the macrophage response and an early indicator of cellular stress. ROS production reflects oxidative stress and is closely linked to inflammatory signaling pathways, including NF-κB activation [67]. Excessive ROS generation can damage lipids, proteins, and DNA, and is often associated with NP-induced cytotoxicity and pro-inflammatory activation. Therefore, ROS measurements provide an important complementary readout to NO for evaluating both immunological activation and oxidative biocompatibility of nanomaterials.

**Figure 7:**
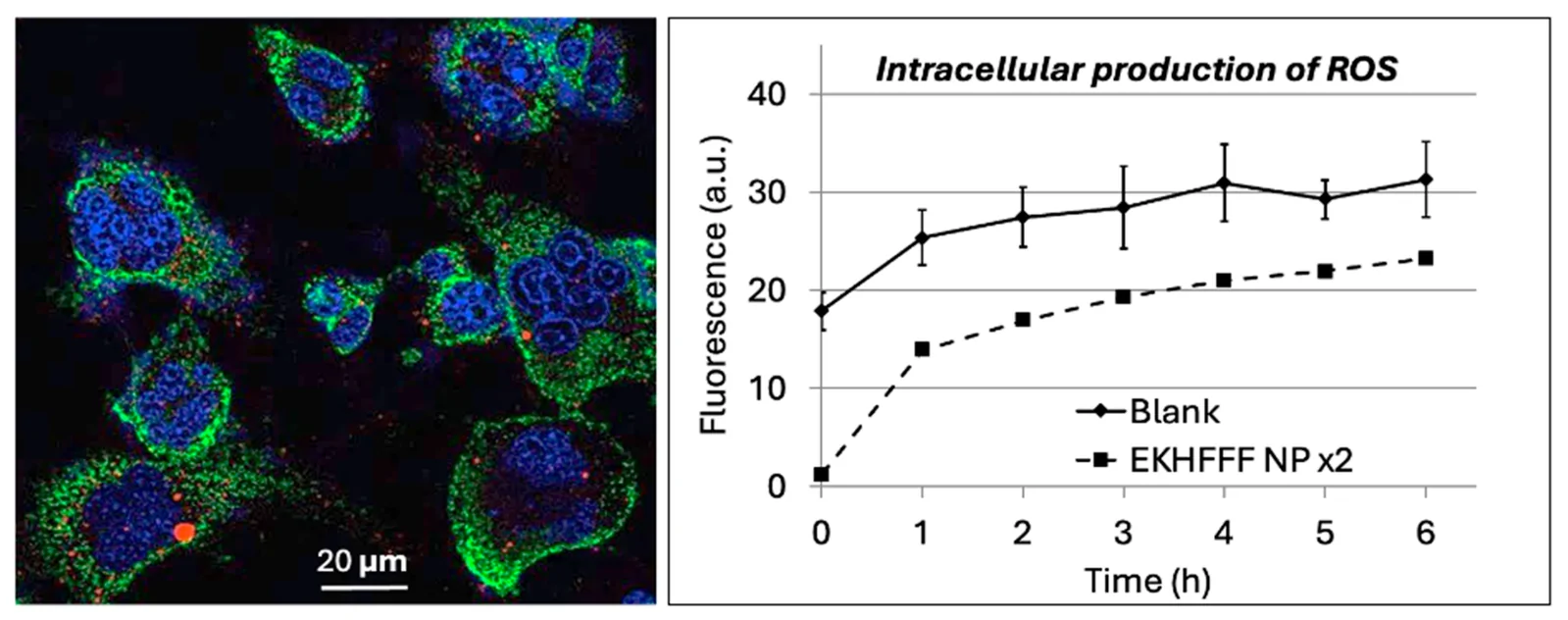
Confocal laser scanning microscopy images showing RAW 264.7 cells following treatment with EKHFFF-Cy5.5 NP. The EKHFFF NP is stained red, nuclei are blue, and the membrane is green (**left**). Intracellular reactive oxygen species (ROS) levels were examined in RAW 264.7 macrophages exposed to EKHFFF NP (**right**). ROS production was monitored for 6 h using the DCFH-DA assay, with fluorescence detection at 480/530 nm. Data are presented as the mean ± SD from three independent experiments (*n* = 3).

Intracellular ROS production was measured using the cell-permeable probe DCFH-DA, which is converted into the highly fluorescent dichlorofluorescein (DCF) in the presence of ROS. Briefly, RAW 264.7 cells were pre-incubated with 20 μM DCFH-DA for 45 min before exposure to varying concentrations of NPs. Fluorescence intensity was measured at 1 h intervals over 6 h using excitation and emission wavelengths of 480 and 530 nm, respectively. The ROS results for the highest NP concentration are presented in Figure 7, while results for all tested concentrations are shown in Figure S8A. Treatment with EKHFFF NPs did not significantly alter ROS production compared with untreated control cells.

Nitrite levels in the culture medium were quantified using the Griess reaction as a measure of NO production. Briefly, RAW 264.7 cells were seeded in 24-well plates and cultured for 24 h before treatment with varying concentrations of EKHFFF NP for an additional 48 h. The PLGA NP was used as a comparison. Subsequently, Griess reagent was added to each well and incubated for 15 min, and absorbance was measured at 550 nm. The results were compared against a 100 ng/mL LPS positive control (PC) and PBS negative control (NC). As shown in Figure S8B, EKHFFF NPs did not induce increased NO production. In contrast, the PLGA NPs led to elevated NO levels, suggesting a protective effect of the EKHFFF coating in mitigating macrophage activation.

Overall, these findings are significant for nanomedicine development, as low or baseline ROS and NO production in RAW 264.7 cells indicates that the EKHFFF NP is biocompatible and is unlikely to trigger acute inflammatory responses, which is critical for in vivo applications.

### 2.4. Evaluating the Therapeutic Effect of EKHFFF-Pt NP

Platinum-based chemotherapy, including cisplatin and carboplatin, remains a standard of care for OvCA. Carboplatin, a second-generation platinum analog, provides a more favorable safety profile with reduced nephrotoxicity, neurotoxicity, and gastrointestinal side effects, as well as more predictable pharmacokinetics that enable easier dosing and improved patient tolerability. The clinical application of platinum-based therapies remains challenging due to dose-limiting systemic toxicity and acquired resistance, emphasizing the need for innovative strategies to enhance drug delivery and therapeutic efficacy. [68,69,70].

To improve platinum delivery, we developed a therapeutic EKHFFF-Pt NP using a PLGA–Pt(II) conjugate synthesized from cisplatin according to our previously reported method [57]. The nanoparticles exhibited a hydrodynamic diameter of 110.4 ± 2.7 nm as determined by DLS and a diameter of 62.0 ± 1.8 nm by TEM (Figure S9). Platinum loading was 0.59% (*w*/*w*), and the zeta potential measured in 10 mM NaCl was −49.1 ± 3.72 mV, confirming the formation of stable, negatively charged nanoparticles. The PLGA–Pt(II) conjugate was further characterized by ^195^Pt NMR (Figure S10, Supplementary Materials).

#### 2.4.1. Stability of EKHFFF NP and EKHFFF-Pt NP in Serum

**Figure 8:**
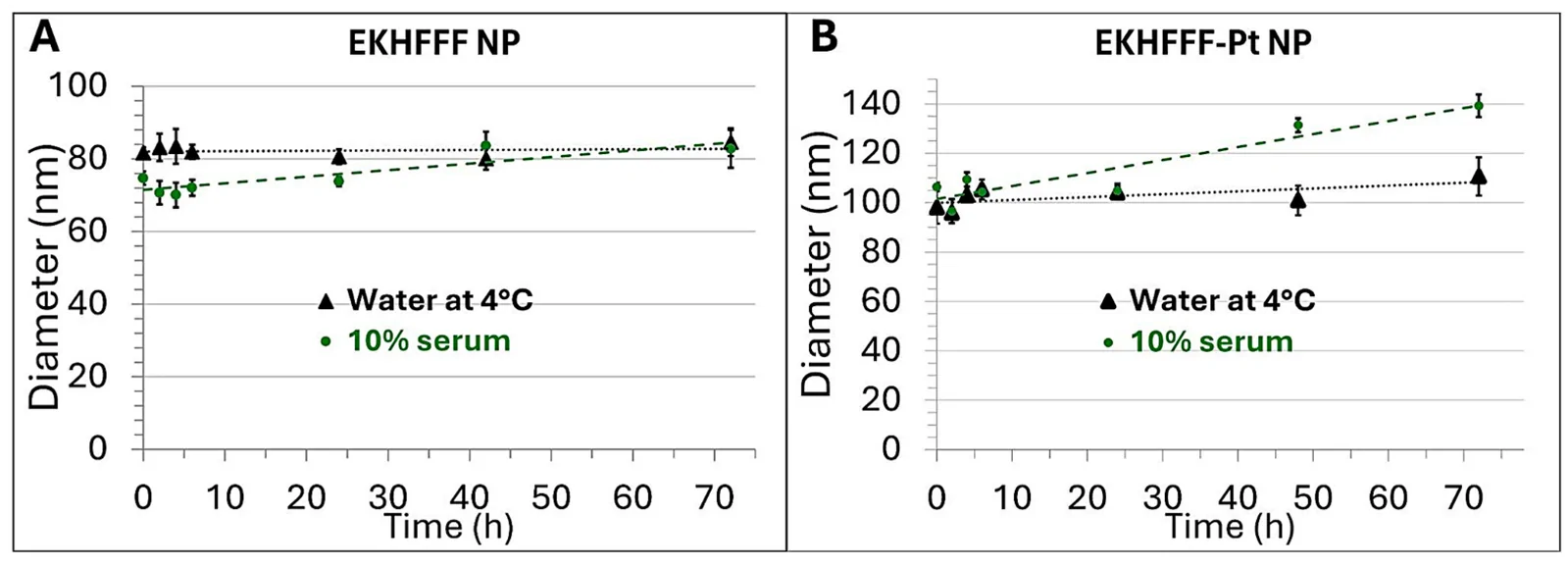
Stability of (**A**) EKHFFF NPs and (**B**) EKHFFF-Pt NPs in 10% serum at 37 °C and in water at 4 °C. Data are presented as mean ± SD from three independent experiments (*n* = 3).

As a preliminary step toward biological studies, we first evaluated the stability of EKHFFF NPs and EKHFFF-Pt NPs under biologically relevant conditions. Briefly, suspensions of NPs (1.2 mg/mL) were prepared in 10% serum and incubated at 37 °C. NP size and polydispersity index (PDI) were monitored over 72 h using dynamic light scattering (DLS). As shown in Figure 8, no significant changes in particle size or PDI (Figures S11 and S12) were observed during the incubation period, indicating good colloidal stability and limited serum-induced aggregation. These results suggest that both EKHFFF NPs and EKHFFF-Pt NPs exhibit sufficient colloidal stability under biologically relevant conditions, supporting their further evaluation in vivo.

#### 2.4.2. Biological Activity of EKHFFF-Pt NP in OvCA Cells

**Figure 9:**
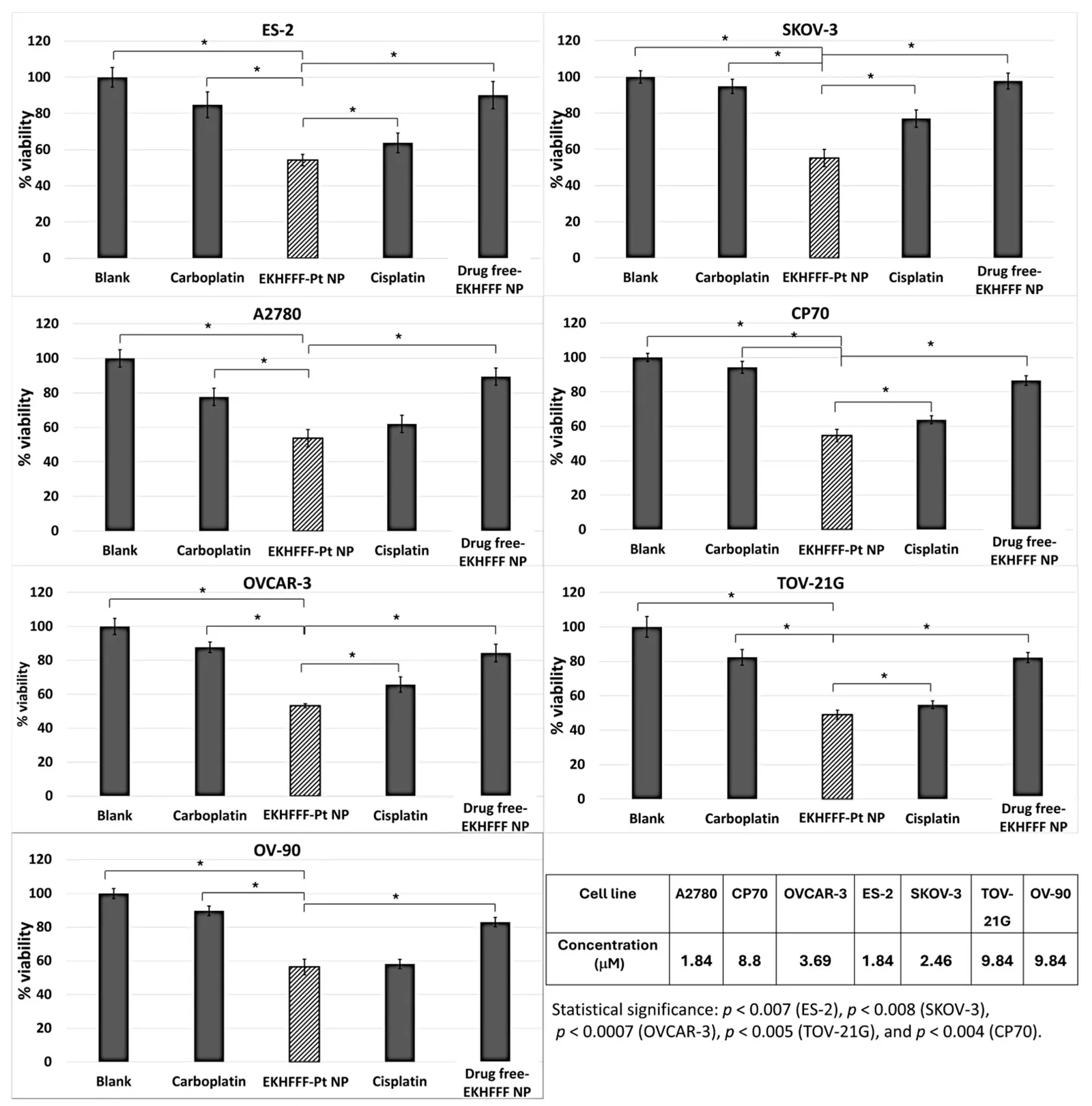
Cytotoxic effects of EKHFFF-Pt NP were evaluated in platinum-sensitive and platinum-resistant OvCA cell lines. Cell viability was determined via MTT assay after 72 h of treatment. EKHFFF-Pt NP, cisplatin, and carboplatin were tested at equivalent Pt(II) concentrations. Data are expressed as mean ± SD based on three independent experiments (*n* = 3). Statistical significance was assessed using Student’s *t*-test. Asterisks (*) indicate statistically significant differences between indicated groups.

Next, the therapeutic efficacy of EKHFFF-Pt NPs was evaluated in OvCA cell cultures comprising both platinum-sensitive and platinum-resistant lines. Cell viability was assessed using the MTT assay and compared with clinically used platinum drugs, including cisplatin and carboplatin, drug-free EKHFFF NPs, and untreated cells. Equivalent Pt(II) concentrations were used for EKHFFF-Pt NPs, cisplatin, and carboplatin in all experiments. Cells were plated in 96-well plates and exposed to EKHFFF-Pt NP for 72 h using Pt(II) concentrations based on the experimentally determined IC_50_ values. The cytotoxicity results are presented in Figure 9.

In platinum-sensitive cell lines, treatment with EKHFFF-Pt NPs, cisplatin, and carboplatin reduced cell viability by 45.8%, 36.3%, and 15.2% (ES-2); 46.3%, 38.0%, and 22.3% (A2780); and 51%, 45.2%, and 17.7% (TOV-21G), respectively, relative to untreated cells after 72 h. A similar trend was observed in platinum-resistant cell lines, with viability reductions of 45.4%, 36.2%, and 5.7% (CP70); 43.6%, 41.8%, and 10.3% (OV-90); 44.8%, 23%, and 5.2% (SKOV-3); and 46.7%, 34.3%, and 12.3% (OVCAR-3) for EKHFFF-Pt NPs, cisplatin, and carboplatin, respectively. Overall, the EKHFFF-Pt NP formulation demonstrated greater cytotoxicity than carboplatin across all cell lines and, in several cases (ES-2, SKOV-3, CP70, OVCAR-3, and TOV-21G), exceeded the activity of cisplatin. Control groups treated with plain EKHFFF NPs showed viability comparable to untreated cells.

#### 2.4.3. In Vivo Tumor Accumulation of EKHFFF-Pt NPs

Next, we evaluated the tumor accumulation potential of EKHFFF-Pt NPs in a preclinical OvCA model as part of a pilot proof-of-concept study. In vivo biodistribution was evaluated using Cy7-labeled EKHFFF-Pt NPs and an IVIS small-animal imaging system, with saline-treated mice used as controls.

**Figure 10:**
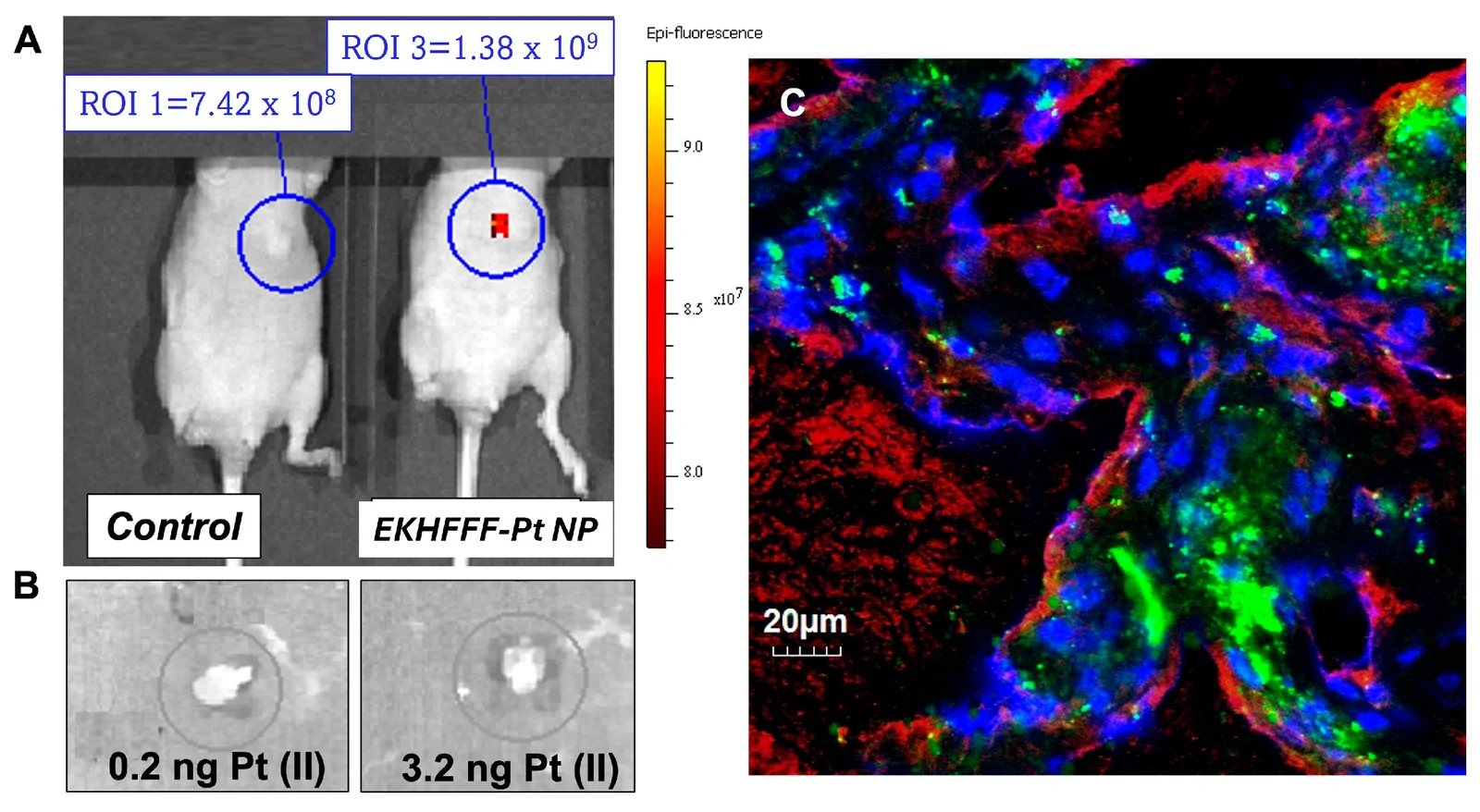
In vivo tumor accumulation and retention of fluorescent EKHFFF-Pt NP in an OvCA model. (**A**) Near-infrared (NIR) imaging at 24 h post injection shows no detectable signal in control (saline-treated) mice, while strong fluorescence is observed at the tumor site in mice treated with fluorescent EKHFFF-Pt NP. (**B**) Quantification of Pt(II) in excised tumors by atomic absorption spectroscopy (AAS) four days post injection. Data are presented as the mean ± SD (*n* = 2). (**C**) Fluorescence imaging of tumor cross-sections; the fluorescent EKHFFF-Pt NP is stained green, tumor blood vessels are red, and the nuclei are blue. Scale bars: 20 µm.

At 24 h following administration, no measurable fluorescence was observed in the control group, whereas animals receiving fluorescently labeled EKHFFF-Pt NPs displayed a pronounced NIR signal localized to the tumor region (Figure 10A), suggesting enhanced nanoparticle localization within the tumor. Furthermore, four days post injection, Pt (II) levels in excised tumors were quantified by atomic absorption spectroscopy (AAS). Tumors from NP-treated mice contained 3.2 ng of Pt(II), demonstrating sustained NP retention within the tumor microenvironment (Figure 10B). Additionally, staining of a tumor cross-section (Figure 10C) revealed the fluorescent EKHFFF-Pt NP (green) present abundantly in the tumor, confirming successful translocation of the NP into the tumor interstitium.

#### 2.4.4. Half-Life and Biodistribution of EKHFFF-Pt NP

Finally, we evaluated the pharmacokinetic profile of the EKHFFF-Pt NP by quantifying its concentration in blood over time using atomic absorption spectroscopy (AAS). The NP was intravenously administered to nude mice at the dose used for the biodistribution study, with blood samples collected at 5, 10, 30, and 90 min after injection. Exponential decay analysis of the concentration-time profile indicated an in vivo half-life of approximately 35 min for the EKHFFF-Pt NP.

The biodistribution of EKHFFF–Pt nanoparticles was evaluated by near-infrared fluorescence (NIRF) imaging, with representative images provided in the Supplementary Materials (Figure S13, left panel). Quantitative biodistribution was further assessed by measuring Pt(II) concentrations in major organs (Figure S13, right panel). Pt(II) accumulation was highest in the spleen and liver, consistent with physiological uptake by the mononuclear phagocyte system. Substantial Pt(II) accumulation was also detected in tumors, demonstrating successful tumor accumulation and retention.

## 3. Materials and Methods

### 3.1. Reagents

Chemicals purchased from Alfa Aesar (Ward Hill, MA, USA) included ninhydrin 99%; N,N′-dicyclohexylcarbodiimide (DCC) 99%; 4-dimethylaminopyridine (DMAP) 99%. Lumiprobe (Cockeysville, MD, USA): Cy5.5 NHS ester, Cy7 NHS ester. Acros Organics (Geel, Belgium): silver nitrate, cis-dichlordiamine platinum (II) 99%; Fisher Chemical (Fair Lawn, NJ, USA): phenol, sodium hydroxide, phosphotungstic acid; PolySciTech (West Lafayette, IN, USA): poly(D,L-lactide-co-glycolide) (PLGA) LA: GA (50:50) mol wt. 5000–10,000 and 24,000–38,000.

Chemicals used for peptide synthesis via the SPPS method were obtained from the following suppliers: Chem-Impex International Inc. (Wood Dale, IL, USA)—Fmoc-L-Phe 4-alkoxybenzyl alcohol resin (0.388 meq/g), Fmoc-L-His-(Boc)OH (99.5%), Fmoc-Glu-OH (99.37%), Fmoc-L-Lys-(Boc)-OH (99.8%), and triisopropylsilane (TIPS); AnaSpec Inc. (Fremont, CA, USA)—2-(1H-benzotriazole-1-yl)-1,1,3,3-tetramethyluronium tetrafluoroborate (TBTU); Alfa Aesar (Ward Hill, MA, USA)—piperidine (99%) and trifluoroacetic acid (TFA, 99%); and Oakwood Chemical (Estill, SC, USA)—N,N-diisopropylethylamine (DIPEA, 99.5%).

Solvents used in the syntheses were obtained from different suppliers and used without further purification. Fisher Chemical (Fair Lawn, NJ, USA) supplied ACS-grade methylene chloride, methanol, chloroform, hexanes, ethyl acetate, 2-propanol (IPA), N,N-dimethylformamide (DMF), and anhydrous ethyl ether. HPLC-grade acetonitrile was purchased from Acros Organics (Geel, Belgium), anhydrous tetrahydrofuran (THF) from Alfa Aesar (Ward Hill, MA, USA), and 190-proof ethyl alcohol from Koptec (King of Prussia, PA, USA).

### 3.2. Instruments

NMR: Bruker Avance III HD spectrometer with a Bruker 400 MHz Ultrashield magnet (Bruker BioSpin Corporation, Billerica, MA, USA).

LC/MS: Agilent Technologies 1200 Series Accurate-Mass TOF LC/MS 6220 (Agilent Technologies, Santa Clara, CA, USA).

HPLC: Flexar High-Performance Liquid Chromatography System (PerkinElmer, Waltham, MA, USA).

DLS: NanoBrook 90Plus PALS (Brookhaven Instruments Corporation, Holtsville, NY, USA).

TEM: FEI Titan Themis 200 (FEI Company, Hillsboro, OR, USA).

Plate reader: SpectraMax M3 (Molecular Devices, San Jose, CA, USA).

Confocal microscope: LSM 880 with Airyscan (Carl Zeiss Microscopy GmbH, Jena, Germany).

Flow cytometer: BD LSR II flow cytometer (BD Biosciences, San Jose, CA, USA).

IVIS: IVIS-200 System (Xenogen Corporation, Alameda, CA, USA).

AAS: AAnalyst 800 (PerkinElmer, Waltham, MA, USA)

### 3.3. Experimental Methods

**Synthesis of the EKHFFF hexapeptide.** The EKHFFF peptide was prepared using a conventional solid-phase peptide synthesis (SPPS) protocol. Fmoc-L-Phe 4-alkoxybenzyl alcohol resin (0.5 g, 0.388 mmol/g) was first swollen in 25.0 mL DMF for 1 h. Fmoc deprotection was carried out using 20% piperidine in DMF, initially for 5 min and subsequently for 20 min. The resin was then subjected to sequential 1 min washes with DMF and IPA in the following order: DMF, IPA, DMF, IPA, DMF, IPA, DMF, and DMF. Successful deprotection and the presence of free NH_2_ groups were verified by the Kaiser test. Following a positive result, Fmoc-L-phenylalanine was coupled to the resin overnight.

Each subsequent amino acid was incorporated using the same coupling procedure. For 0.19 mmol of resin, 0.38 mmol of the appropriate Fmoc-protected amino acid was combined with TBTU (122.3 mg, 0.38 mmol) and DIPEA (98.4 mg, 0.76 mmol) in DMF. After each coupling step, the resin was washed for 1 min sequentially with DMF, IPA, DMF, and IPA, and coupling completion was evaluated using the Kaiser test. Once the peptide sequence was assembled, the resin was subjected to successive 1 min washes with 5 mL each of DMF, IPA, DMF, methanol, dichloromethane, and diethyl ether.

The EKHFFF peptide was released from the resin by treatment with TFA/TIPS/H_2_O (95:2.5:2.5, *v*/*v*/*v*) for 3 h. The resulting crude product was precipitated in cold diethyl ether, washed three times with cold ether, and dried under vacuum. It was subsequently washed three times with 10% HCl and dried again.

Purification was performed by HPLC using an Agilent ZORBAX StableBond 300 C8 column (9.4 × 250 mm, 5 µm; Agilent Technologies, Santa Clara, CA, USA), while a BIOMAP AdvanceBio column (2.7 µm, 3.0 × 150 mm; Agilent Technologies, Santa Clara, CA, USA) was used for analytical evaluation. Chromatographic separation employed an acetonitrile/water mobile-phase system containing 0.01% TFA, with a 10–99% B gradient at 35 °C and a flow rate of 0.5 mL/min.

**X-Ray Photoelectron Spectroscopy (XPS) Analysis.** Characterization of EKHFFF NPs was performed at the Surface Science Facility within the Advanced Science Research Center (ASRC) of The City University of New York (CUNY) using VersaProbe II X-ray photoelectron spectrometer (Physical Electronics, Chanhassen, MN, USA). An aliquot of the nanoparticle suspension was deposited onto a silicon (Si) wafer, dried under vacuum, and subjected to XPS analysis. Survey spectra and high-resolution C1s and N1s spectra were acquired to evaluate the surface elemental composition and confirm the presence of the peptide coating. High-resolution C1s and N1s spectra were analyzed by curve fitting using MultiPak version 9.6.0 (ULVAC-PHI, Chigasaki, Japan) to resolve the individual chemical-state components. A clean Si wafer was analyzed under identical conditions as the control.

**Zeta potential measurement.** Zeta potential and size measurements were performed using DLS. Briefly, 1.2 mg/mL solution of EKHFFF NP in 10.0 mM NaCl was adjusted to the desired pH values by adding 0.1 M HCl or 0.1 M NaOH to decrease or increase the pH, respectively. For ionic strength studies, EKHFFF NP (1.2 mg/mL) and uncoated PLGA NP were dispersed in monovalent salt (NaCl, 1–300 mM) and divalent salt (CaCl_2_, 1–10 mM), and size was measured by DLS. Data are expressed as the mean ± SD.

**Pt(II) release study.** EKHFFF-Pt nanoparticles (20.0 µL) were suspended in 80.0 µL of the corresponding buffer solution in mini dialysis cups (Slide-A-Lyzer MINI Dialysis Units—Thermo Scientific, Waltham, MA, USA). Samples for each time point were prepared in triplicate and incubated at 37 °C for 72 h. Samples were collected at predefined intervals (2, 4, 6, 12, 24, 48, and 72 h), followed by lyophilization and quantification of Pt(II) using atomic absorption spectroscopy (AAS, AAnalyst 800 PerkinElmer, Waltham, MA, USA). Cumulative Pt(II) release was calculated relative to the total amount of Pt(II) initially loaded into the nanoparticles and expressed as a percentage. The results are presented as the mean ± SD.

**Turbidity measurements**. The EKHFFF NP (1.2 mg/mL) and uncoated PLGA NP were dispersed in monovalent salt (NaCl, 50–300 mM) and divalent salt (CaCl_2_, 1–10 mM), and changes in transmittance over time (20 min) were measured using a SpectraMax M3 plate reader (Molecular Devices, San Jose, CA, USA). The results are presented as the mean ± SD.

**The synthesis of plain PLGA NPs and EKHFFF NPs.** To synthesize PLGA NPs or EKHFFF NPs, 5.0 mg (0.0005 mmol) of 5–10 kDa PLGA in 2 mL acetonitrile was added dropwise using a syringe pump (0.2 mL/min flow, (Braintree Scientific, Braintree, MA, USA) to 6 mL of water or to 6.0 mL of water with 1.0 mg (0.0012 mmol) of dissolved EKHFFF at 37 °C. The NP suspensions were subsequently stirred overnight. After 24 h, the NPs were concentrated using a Vivaspin centrifugal concentrator (100 kDa MWCO; Sartorius, Göttingen, Germany) and washed three times with water.

**Synthesis of the PLGA–Pt(II) Conjugate.** Step 1. Activation of Pt(II). Cisplatin (50.0 mg, 0.167 mmol) was suspended in hot water (4.0 mL). A solution of silver nitrate (55.2 mg, 0.325 mmol) in water (0.5 mL) was added dropwise, and the reaction mixture was stirred at 60 °C for 2 h. Completion of the activation reaction was confirmed by the absence of Ag^+^ ions using a 10% HCl test. The AgCl precipitate formed was separated by centrifugation, after which the supernatant was recovered, passed through a 0.2 μm syringe filter, and analyzed for Pt(II) using SnCl_2_. The resulting filtrate containing activated Pt(II) was used directly in the subsequent conjugation step. Step 2. Conjugation of activated Pt(II) to PLGA. Sodium hydroxide (13.3 mg, 0.333 mmol) was dissolved in water (3.0 mL) at 60 °C, followed by the addition of PLGA (100.0 mg, 0.013 mmol). The activated Pt(II) solution obtained in Step 1 was then added, and the reaction mixture was stirred at 60 °C for 2 h, followed by overnight stirring at room temperature. The resulting brown-yellow product was collected by centrifugation and concentrated using a Rotavapor R-3 (BÜCHI Labortechnik AG, Flawil, Switzerland). The crude product was dissolved in a minimal volume of acetonitrile (ACN) and precipitated by addition to cold methanol. The purified PLGA–Pt(II) conjugate was collected and dried prior to further use. Step 3. ^195^Pt NMR Analysis of the PLGA–Pt(II) Conjugate. The PLGA–Pt(II) conjugate was hydrolyzed in an approximately 1:1 (*v*/*v*) mixture of acetone and 10% aqueous HCl for 72 h at 35 °C under stirring. After removal of insoluble PLGA residue by centrifugation, the aqueous phase was lyophilized, and the residue was reconstituted in D_2_O for ^195^Pt NMR analysis. Spectra were acquired in the chemical shift regions characteristic of Pt(II) (approximately −2000 ppm) and Pt(IV) (approximately +900 ppm). A signal was observed exclusively in the Pt(II) region, consistent with square-planar Pt(II) complexes such as cisplatin, while no signal was detected in the Pt(IV) region, confirming the absence of detectable Pt(IV) species. The spectral width for both acquisitions was approximately 2500 ppm. Data acquisition and processing were carried out using TopSpin 3.5 software.

**The synthesis of EKHFFF-Pt NP**. EKHFFF-Pt NPs were prepared via nanoprecipitation of the PLGA-Pt hybrid in the presence of the EKHFFF peptide. A solution of PLGA-Pt (5.0 mg, 0.0012 mmol) in acetonitrile (2.0 mL) was introduced into an aqueous EKHFFF solution (1.0 mg, 0.0012 mmol in 6.0 mL water) at 37 °C using a syringe pump (Braintree Scientific, Braintree, MA, USA) at a flow rate of 0.2 mL/min. The resulting dispersion was maintained under continuous stirring overnight to promote nanoparticle self-assembly. The formed EKHFFF-Pt NPs were subsequently concentrated using a Vivaspin centrifugal concentrator (100 kDa MWCO; Sartorius, Göttingen, Germany) and purified by three consecutive washes with water.

**The synthesis of PLGA-Cy5.5 and PLGA-Cy7 hybrid.** PLGA conjugates with Cy5.5 or Cy7 were prepared by reacting 200.0 mg (0.006 mmol) of 24–38 kDa PLGA with either Cy5.5 NHS ester (9.7 mg, 0.0097 mmol) or Cy7 NHS ester (7.0 mg, 0.0097 mmol). DCC (2.66 mg, 0.013 mmol) and DIPEA (3.33 mg, 0.026 mmol) were subsequently added, and all components were dissolved in DMF. The reaction was allowed to proceed overnight at 4 °C under gentle stirring. Following solvent removal by rotary evaporation using a Rotavapor R-3 (BÜCHI Labortechnik AG, Flawil, Switzerland), the crude material was redissolved in approximately 1 mL of acetonitrile and precipitated by dropwise addition into cold methanol. The mixture was stored at −20 °C overnight, followed by centrifugation and redissolution of the recovered product in acetonitrile. This precipitation–centrifugation procedure was performed three times to purify the conjugate. The final product was lyophilized, yielding a dark-blue solid. The reaction yields were 70% for PLGA-Cy5.5 and 72% for PLGA-Cy7.

**The synthesis of EKHFFF-Cy5.5 NPs and EKHFFF-Cy7 NPs.** To synthesize EKHFFF-Cy5.5 NPs and EKHFFF-Cy7 NPs, 4.0 mg (0.0006 mmol) of plain 5–10 kDa PLGA and 1.0 mg (0.0000323 mmol) of PLGA-Cy5.5 or PLGA-Cy7 in 2 mL acetonitrile were added dropwise using a syringe pump (0.2 mL/min flow, Braintree Scientific, Braintree, MA, USA) to 6 mL of water with 1.0 mg (0.0012 mmol) of dissolved EKHFFF at 37 °C. Next, the solution was stirred overnight to allow for the self-assembly of EKHFFF-Cy5.5 NPs or EKHFFF-Cy7 NPs. After that the NPs were concentrated in Vivaspin centrifugal concentrator (100 kDa MWCO; Sartorius, Göttingen, Germany) and washed 3-times with water.

**Confocal imaging and 3D Imaris reconstruction.** A2780, CP70, SKOV-3, OV-90, TOV-21G, ES-2, OVCAR-3, and RAW 264.7 cells were seeded in 3 × 10^4^ cells per chamber in four-chamber, confocal-ready plates Lab-Tek II Chamber Slide (Cat. No. 154536, Thermo Fisher Scientific, Rochester, NY, USA). Following 24 h of culture, cells were exposed to EKHFFF-Cy5.5 NP (0.25 mg/mL) for an additional 48 h. The cells were subsequently rinsed twice with PBS and stained with WGA–Alexa Fluor 488 (10 μg/mL) for 15 min at 37 °C. After two additional PBS washes, fixation was performed with 4% PFA for 20 min at 37 °C. Finally, the cells were mounted using DAPI-containing mounting medium and visualized with an LSM 880 Airyscan Fast Live Cell confocal microscope (Carl Zeiss Microscopy, Jena, Germany). Confocal Z-stack images of ES-2 cells treated with EKHFFF-Cy5.5 NPs, as described above, were acquired and imported into Imaris software (version 9.7; Bitplane, Oxford Instruments, Abingdon, UK) for 3D reconstruction.

**Flow Cytometry (FACS).** OvCA cells and PDCLs were seeded at 3.6 × 10^5^ cells/well in 6 well plates. After 48 h, 100.0 μL of EKHFFF-Cy5.5 NP in fresh media were added to each well and incubated for 15 min. Next, the cells were washed 3-times with PBS and fixed with 1% of paraformaldehyde. Cells were maintained at 4 °C overnight, followed by fluorescence analysis using a BD LSR II flow cytometer (BD Biosciences, Franklin Lakes, NJ, USA).

**ROS studies.** RAW 264.7 murine macrophages (ATCC, Manassas, VA, USA) were plated in black 96-well plates with clear bottoms at a density of 5 × 10^4^ cells per well and cultured in standard DMEM supplemented with 10% serum. Following 24 h of culture, the cells were rinsed three times with PBS and loaded with 100.0 μL of 20.0 μM DCFH-DA (2′,7′-dichlorofluorescein diacetate; MilliporeSigma, Burlington, MA, USA, 287810) per well. After incubation for 45 min at 37 °C under 5% CO_2_, excess dye was removed by three PBS washes. The cells were then treated with EKHFFF NP prepared at different concentrations in serum-free medium (Gibco RPMI 1640 1X; 11835-030; Thermo Fisher Scientific, Waltham, MA, USA). Fluorescence intensity was monitored hourly over a 6 h period at excitation and emission wavelengths of 480 and 530 nm, respectively. The tested EKHFFF NP concentrations were 0.0255 mg/mL (×1.5), 0.017 mg/mL (×1), 0.0034 mg/mL (×0.2), and 0.00068 mg/mL (×0.04). LPS (100.0 ng/mL) served as the positive control (PC), whereas untreated RAW 264.7 cells exposed to PBS were used as the negative control (NC). The ×1 NP concentration (0.017 mg/mL) represented the theoretical plasma drug concentration estimated from the corresponding human cisplatin dose.

**RNS studies.** The experiment was performed according to the NIH Nanotechnology Characterization Laboratory protocol (NCL Method ITA-7). RAW 264.7 cells were maintained in complete RPMI-1640 medium (Gibco, Thermo Fisher Scientific, Waltham, MA, USA) containing 10% heat-inactivated fetal bovine serum with L-glutamine (HyClone, Logan, UT, USA), β-mercaptoethanol, and penicillin/streptomycin (HyClone, Logan, UT, USA) under standard culture conditions (37 °C, 5% CO_2_, humidified atmosphere).

For sample exposure, RAW 264.7 cells were plated in 24-well plates at 2 × 10^6^ cells per well and allowed to equilibrate overnight. The appropriate test samples, controls, and blanks were then introduced into the corresponding wells, followed by incubation for 48 h under the same culture conditions. Following treatment, 50.0 μL aliquots from each culture well were transferred in duplicate to a 96-well assay plate. Quality controls and calibration standards prepared in culture medium were included in four replicate wells for each concentration. Griess reagent (100.0 μL) was added to each well, and the plate was mixed for 2 min before measuring absorbance at 550 nm. EKHFFF NP dosing was based on the theoretical plasma concentration corresponding to a cisplatin-equivalent human dose, with 0.01365 mg/mL defined as the 1× concentration.

**Transmission electron microscopy (TEM) imaging of the EKHFFF NP and EKHFFF-Pt NP.** The NP samples were mixed with the acetate buffer (0.125 M CH_3_COONH_4_, 0.6 mM (NH_4_)_2_CO_3_ and 0.26 mM tetrasodium EDTA at pH 7.4). A 10.0 μL aliquot of the sample was mixed with an equal volume of 2% (*w/v*) phosphotungstic acid for negative staining. The mixture was deposited onto a 200-mesh carbon-coated copper grid (Electron Microscopy Sciences, Hatfield, PA, USA) and allowed to air-dry. TEM images were acquired using an FEI Titan Themis 200 transmission electron microscope (FEI Company, Hillsboro, OR, USA).

**Stability of NPs measured by dynamic light scattering (DLS).** A total of 100.0 μL of the NP solution was mixed with 2900.0 μL of nanopure water (stored at 4 °C) or 10% fetal bovine serum (FBS, incubated at 37 °C). The hydrodynamic diameter was measured by DLS, and the average particle size and polydispersity index (PDI) were calculated from three independent measurements.

### 3.4. In Vitro Experiments

**Cell culture methods.** A2780, CP70, SKOV-3, OV-90, TOV-21G, ES-2, and OVCAR-3 cell lines were obtained from ATCC, Manassas, VA, USA. Cells were maintained in Dulbecco’s Modified Eagle Medium (Sigma-Aldrich, St. Louis, MO, USA) supplemented with fetal bovine serum (FBS, HyClone, Logan, UT, USA), L-glutamine (HyClone, Logan, UT, USA), and penicillin/streptomycin (HyClone, Logan, UT, USA,. The medium contained 10% FBS for A2780, CP70, SKOV-3, ES-2, and OVCAR-3 cells and 15% FBS for TOV-21G and OV-90 cells. Cultures were maintained at 37 °C in a humidified atmosphere containing 5% CO_2_. For in vitro experiments, cells were plated in 96-well plates at a density of 3 × 10^5^ cells per well and allowed to adhere overnight before treatment. EKHFFF NP suspensions were prepared directly in the corresponding growth medium. Stock solutions of cisplatin/carboplatin were prepared in the growth medium. For all cytotoxicity experiments, the concentrations of EKHFFF-Pt NP, cisplatin, and carboplatin were normalized to an equivalent Pt(II) concentration. Cells were exposed to the treatment for 72 h, after which cell viability and cytotoxicity were assessed using the TACS MTT Cell Proliferation Assay (Trevigen, Gaithersburg, MD, USA) according to the manufacturer’s protocol. Absorbance was measured using a SpectraMax M3 plate reader (Molecular Devices, San Jose, CA, USA).

### 3.5. In Vivo Experiments

Approval for all in vivo experimental procedures was obtained from the Institutional Animal Care and Use Committee (IACUC) at the Icahn School of Medicine at Mount Sinai. Mice were maintained under standard husbandry conditions at the Center for Comparative Medicine and Surgery, with unrestricted access to food and water, in compliance with the Guide for the Care and Use of Laboratory Animals. Animals were monitored twice daily for general health throughout the study. Body weight and tumor growth were assessed once weekly. No unexpected adverse events were observed during the study. Five-week-old female nude mice (Charles River Laboratories, Wilmington, MA, USA), weighing 18.0–20.0 g, were maintained under specific pathogen-free conditions and used to establish the ovarian cancer tumor model and for in vivo half-life studies. The EKHFFF-Pt NP was administered via tail vein injection to OVCAR-3 tumor-bearing nude mice at a dose of 0.314 mg Pt(II), corresponding to a clinically relevant, bioequivalent cisplatin dose (75 mg/m^2^).

**In vivo fluorescence imaging.** Near-infrared fluorescence (NIRF) imaging was conducted both in vivo and ex vivo using the IVIS-200 System (Xenogen Corporation, Alameda, CA, USA). EKHFFF-Pt-Cy7 NPs were visualized using excitation and emission filters of 745 and 800 nm, respectively. Images were acquired with a field of view (FOV) of 17.6 and an exposure time of 4 s.

**Tumor tissue slide.** Frozen tumor tissues were sectioned using a cryostat and subsequently processed for CD31 immunofluorescence staining and nuclear counterstaining with DAPI. Sections were initially blocked with donkey serum for 20 min at room temperature, followed by washing with Dulbecco’s phosphate-buffered saline (DPBS; Corning, NY, USA). The samples were then incubated for 45 min at room temperature with rat anti-mouse CD31 antibody (BD Pharmingen, Franklin Lakes, NJ, USA; Cat. No. 557355). After PBS washing, Cy3-conjugated donkey anti-rat secondary antibody (Jackson ImmunoResearch Laboratories, Inc., Cat. No. 712-165-150, West Grove, PA, USA) was applied for 30 min. The sections were washed again and mounted using DAPI-containing mounting medium. Fluorescence images were acquired with an LSM 880 Airyscan Fast Live Cell confocal microscope (Carl Zeiss Microscopy GmbH, Jena, Germany) using appropriate fluorescence settings and 40× and 63× objectives.

**Mouse Tumor Model.** An OVCAR-3 xenograft model was generated by subcutaneous injection of 1 × 10^7^ cells into the right hind limb of nude mice (*n* = 2). Tumor growth was monitored by external caliper measurements, and tumor volume was calculated as *V* = *L* × *W^2^* × 0.5, where *L* and *W* represent the longest and shortest tumor diameters, respectively. Once tumors reached approximately 500 mm^3^, the animals were randomly allocated to receive either the EKHFFF-Pt-Cy7 NP or saline as the control treatment. Mice were injected via tail vein with EKHFFF-Pt-Cy7 NP containing 0.314 mg Pt(II), with saline-treated mice (*n* = 2) serving as controls. No animals were excluded during the experiment or data analysis. At 24 h after injection, in vivo fluorescence imaging was performed for all mice, as further described below. Four days after injection, the experiment was terminated. Following the imaging procedure, the mice were euthanized, and the subcutaneous tumors together with the brain, lungs, liver, kidneys, spleen, and heart were harvested for ex vivo fluorescence imaging. No experimental units were excluded from the analysis.

**In vivo fluorescence imaging.** Near-infrared fluorescence (NIRF) imaging of live animals and excised tissues was carried out with the IVIS-200 System (Xenogen Corporation, Alameda, CA, USA). Cy7-labeled NPs were detected using 745 nm excitation and 800 nm emission filters. Images were acquired with a field of view (FOV) of 17.6 and an exposure time of 4 s.

**In vivo half-life and biodistribution of EKHFFF-Pt NPs**. To evaluate the in vivo half-life of the NPs, nude mice (*n* = 2) received the formulation intravenously via the tail vein. Blood samples were subsequently obtained by retro-orbital collection at 5, 10, 30, and 90 min post injection. Platinum concentrations in whole blood were quantified by atomic absorption spectroscopy (AAS, AAnalyst 800 PerkinElmer, Waltham, MA, USA). The circulation half-life was estimated from the decrease in blood Pt(II) concentration over time.

For biodistribution studies, mice were euthanized 4 days after nanoparticle administration, and the tumor, liver, spleen, kidneys, lungs, and heart were harvested, digested in freshly prepared aqua regia (HCl/HNO_3_, 3:1, *v*/*v*), and analyzed for Pt(II) content by AAS, AAnalyst 800 (PerkinElmer, Waltham, MA, USA) to determine nanoparticle biodistribution and tumor accumulation. Organ accumulation was calculated as the amount of Pt(II) per mg of organ tissue.

## 4. Conclusions

This study presents the development and comprehensive evaluation of pH-responsive EKHFFF NPs for ovarian cancer drug delivery. The peptide coating enables tunable surface properties, allowing the NPs to remain stable and negatively charged under physiological conditions while reducing their negative charge in the mildly acidic tumor microenvironment to promote nanoparticle–cell interactions. The NPs exhibit favorable size, stability in serum, and resistance to aggregation under physiologically relevant ionic conditions. In addition, the pH-responsive surface properties were accompanied by enhanced Pt(II) release under mildly acidic conditions, supporting their potential for tumor-associated drug delivery. In vitro studies demonstrated efficient cellular uptake, particularly in PDCL, and minimal induction of inflammatory responses, indicating good biocompatibility. When loaded with Pt (II), the EKHFFF NP demonstrated greater cytotoxicity than carboplatin and, in several ovarian cancer cell lines, comparable or greater activity than cisplatin. In vivo, nanoparticles demonstrated preferential tumor accumulation, sustained intratumoral retention, and a measurable circulation half-life. Collectively, these results support the potential of EKHFFF NPs as a pH-responsive platform for preferential tumor accumulation and platinum-based drug delivery in ovarian cancer.

## 5. Patents

A patent application related to the technology described in this work has been filed with the United States Patent and Trademark Office (USPTO) under U.S. Provisional Patent Application No. 64/048,368.

## Figures and Tables

**Table 1:** Calculated EKHFFF peptide charge and corresponding zeta potential of EKHFFF NP across different pH conditions.

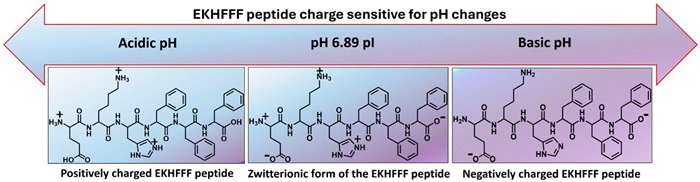							
pH Unit	C_α-_NH_2_ (Glu)	-COOH (Glu)	-NH_2_ (Lys)	-Imidazole (His)	C_α_-COOH (Phe)	Calculated EKHFFF Peptide Charge	Experimental EKHFFF NP Charge (mV)
	pK_b_ 9.67	pK_a_ 4.25	pK_b_ 10.53	pK_b_ 6.00	pK_a_ 3.1		
3	+	+	+	+	0	+4	−11.3 ± 0.98
4	+	0	+	+	−	+2	−26.9 ± 1.42
5	+	−	+	+	−	+1	−33.3 ± 1.47
6	+	−	+	0	−	0	−38.6 ± 2.87
7	+	−	+	0	−	0	−43.2 ± 1.21

## Data Availability

The data presented in this study are available from the corresponding author upon reasonable request. Certain data are not publicly available due to intellectual property and patent protection considerations.

## Supplementary Materials

The following supporting information can be downloaded at: https://www.mdpi.com/article/10.3390/molecules31172953/s1, Figure S1: HRMS-ESI of the EKHFFF peptide: *m*/*z* [M+H]^+^ calc. for C_44_H_55_N_9_O_9_ 853.41; found 854.4185; Figure S2: An HPLC chromatogram of the EKHFFF peptide showing a retention time of 5.40 min; Figure S3. Top: The XPS spectrum of the Si wafer in the binding energy region of N1s shows only noise. Middle: The C1s carbon XPS spectrum of EKHFFF-NPs, fit with three peaks at 284.82 (adventitious carbon, largest peak), 286.92, and 289.21 eV. Bottom: The N1s XPS nitrogen spectrum of EKHFFF-NPs, fit with three peaks at 399.10 (largest peak), 400.22, and 401.18 (smallest peak) eV. Gaussian–Lorentzian line shapes were used for fitting both the C1s and N1s spectra. A linear background was applied to the C1s spectrum, whereas a Shirley background was used for the N1s spectrum. The χ^2^ values were 14.65 for the C1s fit and 1.09 for the N1s fit. Curve fitting was performed using MultiPak software (Version 9.6.0). Figure S4: Elemental analysis reports for (top) PLGA NPs and (bottom) EKHFFF NPs performed by Atlantic Microlab. Figure S5. pH-dependent Pt(II) release profile of EKHFFF-Pt nanoparticles over 72 h at 37 °C. Data are presented as mean ± SD from three independent experiments (*n* = 3). Figure S6: Transmittance kinetics of EKHFFF NP as a function of time at different ionic strengths. Data are presented as mean ± SD from three independent experiments (*n* = 3); Figure S7. Cellular uptake data of EKHFFF-Cy5.5 nanoparticles in ovarian cancer cell lines (OvCA) and patient-derived ovarian cancer cell lines (PDCLs) after 15 min incubation, determined by flow cytometry. Data are presented as mean ± SD from three independent experiments (*n* = 3); Figure S8: (A) Assessment of intracellular reactive oxygen species (ROS) production in RAW 264.7 cells following exposure to EKHFFF NP in different concentrations. (B) Nitrite accumulation in culture supernatants was quantified using the Griess reaction after 48 h exposure to nanoparticles. LPS (100 ng/mL) and PBS were used as positive and negative controls, respectively. Data are presented as mean ± SD from three independent experiments (*n* = 3); Figure S9. The TEM image for EKHFFF-Pt NP. The average nanoparticle size was 62.2 ± 1.08 nm. Data are presented as mean ± SD from three independent experiments (*n* = 3); Figure S10. ^195^Pt NMR spectra of the hydrolyzed PLGA–Pt(II) conjugate: (A) Pt(II) region showing the characteristic Pt(II) signal and (B) Pt(IV) region showing no detectable signal. Spectra were acquired on a Bruker Avance III HD spectrometer equipped with a 400 MHz UltraShield magnet and processed using TopSpin 3.5 software; Figure S11: Polydispersity index (PDI) of EKHFFF NP under different conditions. Data are presented as mean ± SD from three independent experiments (*n* = 3); Figure S12: Polydispersity index (PDI) of EKHFFF-Pt NP under different conditions. Data are presented as mean ± SD from three independent experiments (*n* = 3); Figure S13: NIRF images of organs excised from mice after in vivo experiments (left) and corresponding Pt(II) mass (mg) in the organs (right); Movie S1. Three-dimensional (3D) Imaris reconstruction of ES-2 cells treated with EKHFFF–Cy5.5 nanoparticles. The video is provided as a separate supplementary file.

## Abbreviations

The following abbreviations are used in this manuscript:

DLS	Dynamic Light Scattering
EPR	Enhanced Permeability and Retention
NP	Nanoparticle
pI	Isoelectric point
PLGA	Poly(lactic-co-glycolic) acid
PDI	Polydispersity index
PDCL	Patient-Derived Cell Line
ROS	Reactive Oxygen Species
TEM	Transmission Electron Microscopy
XPS	X-ray Photoelectron Spectroscopy
ZP	Zeta Potential
