# Supplementary material for: pH-Dependent Surface Charge Modulation of Peptide-Coated Poly(lactic-co-glycolic Acid) (PLGA) Nanoparticle for Drug Delivery in Ovarian Cancer
Source: Molecules. 2026 Aug 23;31(17):2953. doi: 10.3390/molecules31172953 (PMC13567042; doi:10.3390/molecules31172953)
Supplement: Supplementary file 1 [file molecules-31-02953-s001.zip › molecules-4391442-supplementary.pdf]

pH-Dependent Surface Charge Modulation of Peptide-Coated Poly(lactic-co-glycolic Acid) (PLGA) Nanoparticle for Drug Delivery in Ovarian Cancer

SUPPLEMENTARY MATERIALS

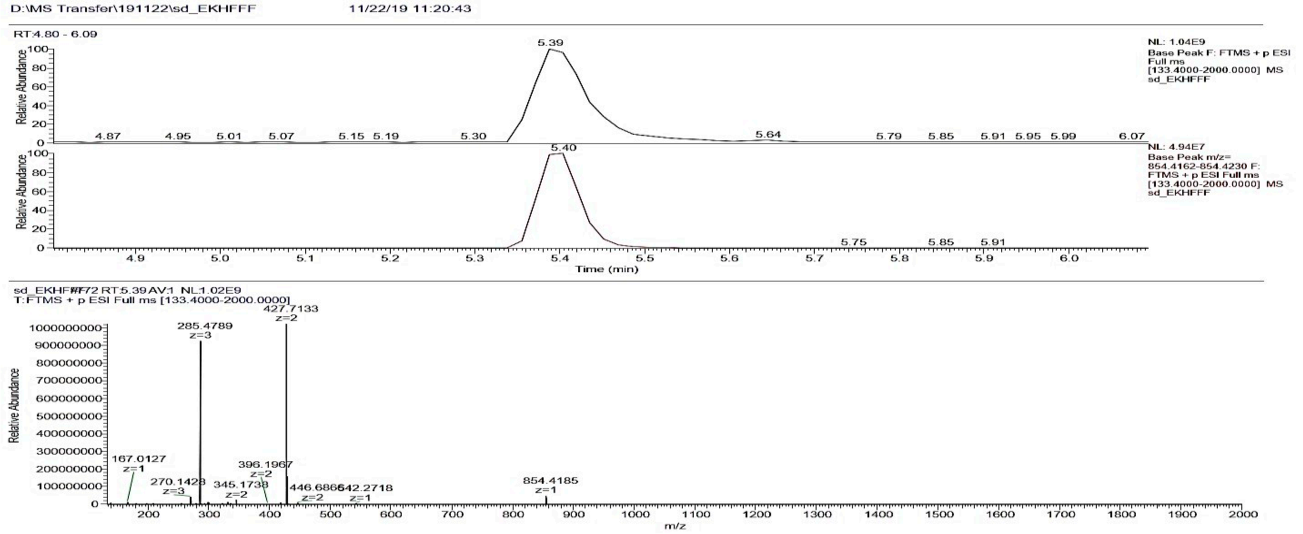

Figure S1. HRMS-ESI of the EKHFFF peptide: m/z $[M+H]^+$ calc. for $C_{44}H_{55}N_9O_9$ 853.41; found 854.4185.

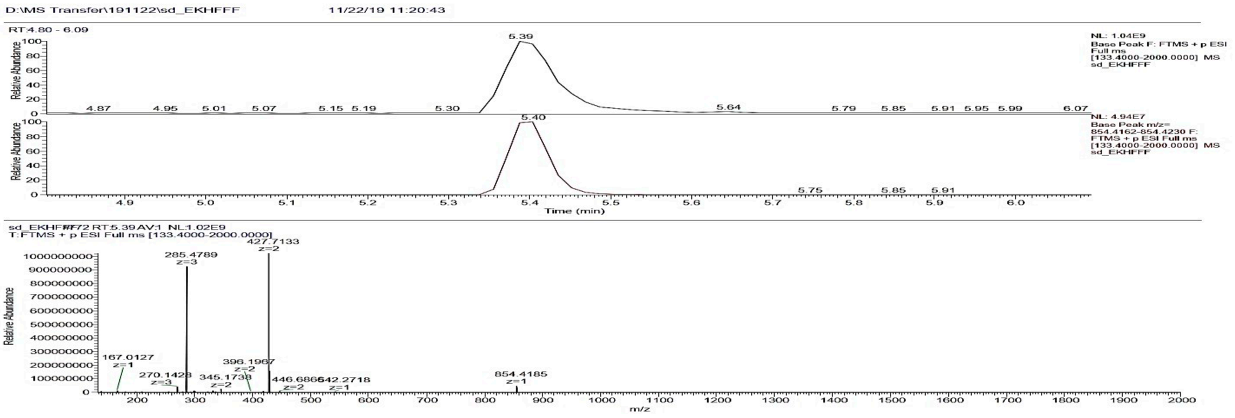

Figure S2. HPLC chromatogram of the EKHFFF peptide showing a retention time of 5.40 min.

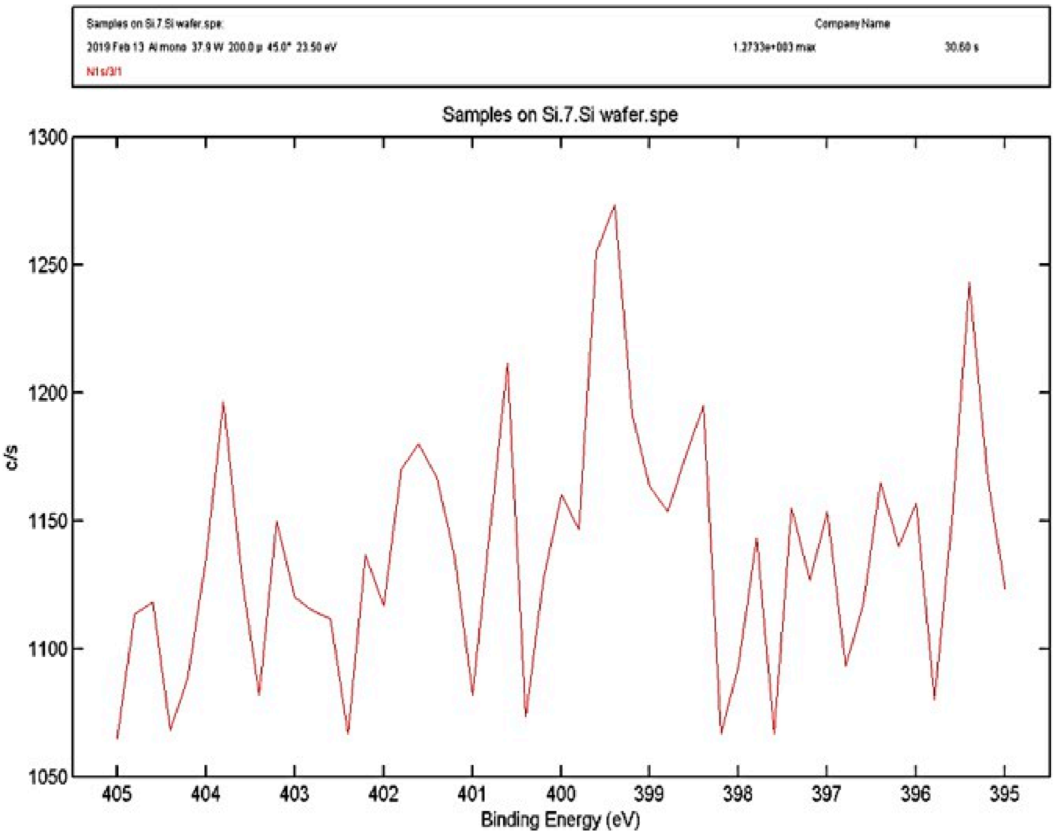
Samples on Si.7.Si wafer.spe:
Company Name
2019 Feb 13 Al mono 37.9 W 200.0 μ 45.0° 23.50 eV
1.3733e+003 max
30.60 s
N1s/3/1
Samples on Si.7.Si wafer.spe
c/s
1300
1250
1200
1150
1100
1050
405
404
403
402
401
400
399
398
397
396
395
Binding Energy (eV)

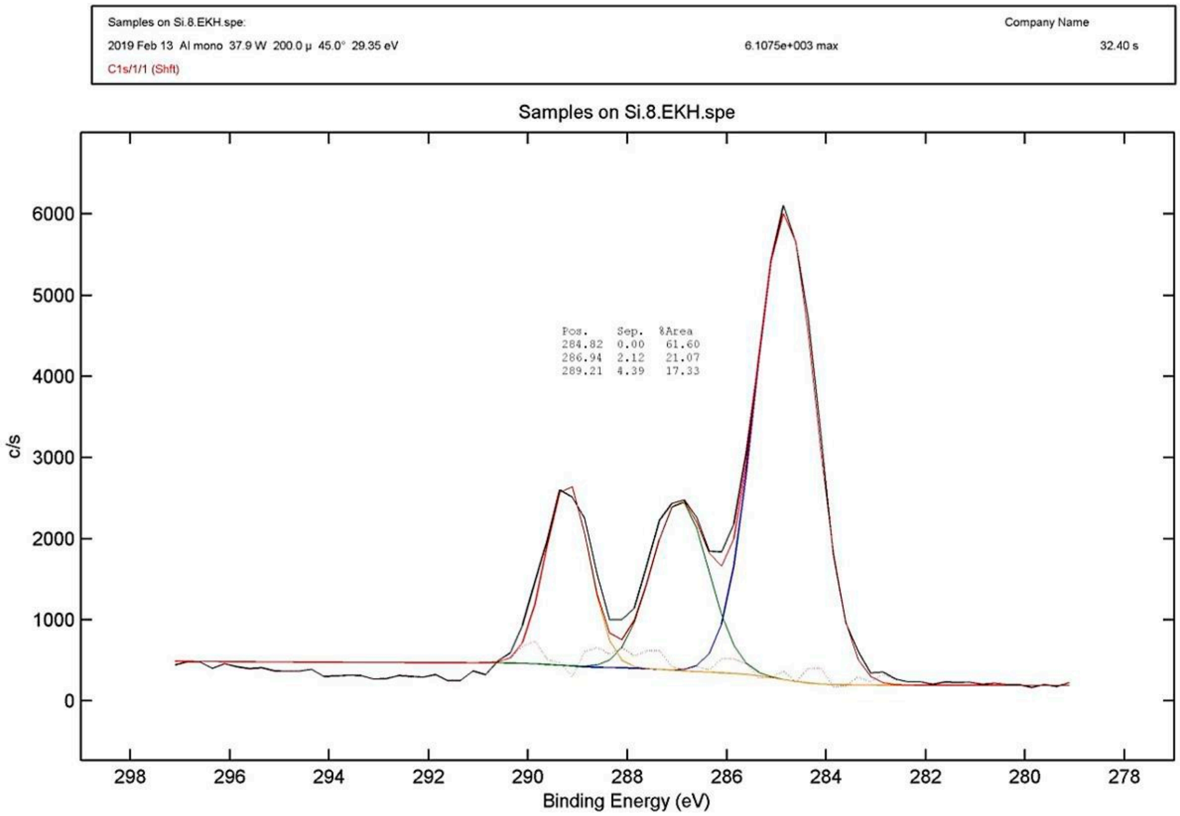
Samples on Si.8.EKH.spe:
Company Name
2019 Feb 13 Al mono 37.9 W 200.0 μ 45.0° 29.35 eV
6.1075e+003 max
32.40 s
C1s/1/1 (Shft)
Samples on Si.8.EKH.spe
Pos. Sep. %Area
284.82 0.00 61.60
286.94 2.12 21.07
289.21 4.39 17.33
c/s
6000
5000
4000
3000
2000
1000
0
298
296
294
292
290
288
286
284
282
280
278
Binding Energy (eV)

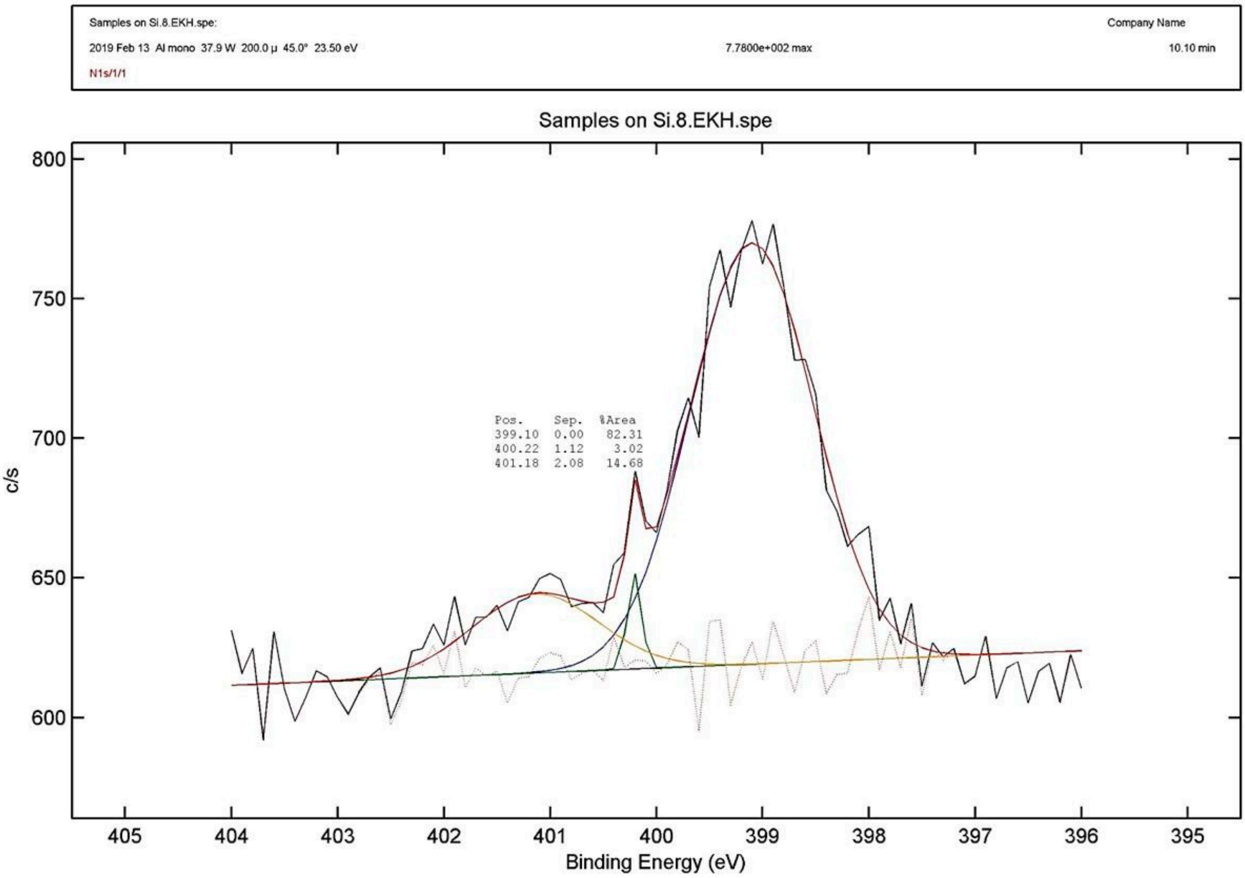

Figure S3. Top: The XPS spectrum of the Si wafer in the binding energy region of N1s shows only noise. **Middle:** The C1s carbon XPS spectrum of EKHFFF-NPs, fit with three peaks at 284.82 (adventitious carbon, largest peak), 286.92, and 289.21 eV. **Bottom:** The N1s XPS nitrogen spectrum of EKHFFF-NPs, fit with three peaks at 399.10 (largest peak), 400.22, and 401.18 (smallest peak) eV. Gaussian–Lorentzian line shapes were used for fitting both the C1s and N1s spectra. A linear background was applied to the C1s spectrum, whereas a Shirley background was used for the N1s spectrum. The χ^2 values were 14.65 for the C1s fit and 1.09 for the N1s fit. Curve fitting was performed using MultiPak software (Version 9.6.0.15).

Atlantic Microlab, Inc.

Sample No. SD-2-083 B (17.9 mg) PLGA NP (uncoated)

6180 Atlantic Blvd. Suite M
Norcross, GA 30071
www.atlanticmicrolab.com

Company/School BROOKLYN COLLEGE
Dept. CHEMISTRY
Address 2900 BEDFORD AVE, ROOM # 350 NE
City, State, Zip BROOKLYN, NY, 11210

Professor/Supervisor: ANETA MIESZAWSKA
PO# / CC# AM06172021

Name SYLWIA DRAGULSKA Date 06/12/2021
Phone (718) 951-5000

Element	Theory	Found	
C	46.13	45.48	45.59
H	4.61	5.00	5.03
N	0.00	0.0	0.0

Single ☐ Duplicate ☒
Elements Present: C, H, O
Analyze for: C, H, N
Hygroscopic ☐ Explosive ☐
M.P. ______ B.P. ______
To be dried: Yes ☐ No ☒
Temp. ______ Vac. ______ Time ______
Rush Service ☐ Rush service guarantees analyses will be completed and results available by 5 PM EST on the day the sample is received by 11 AM.
Include Email Address or FAX # Below
sylwia.dragulska@brooklyn.cuny.edu

Date Received JUN 2 1 2021 Date Completed JUN 2 2 2021

Remarks: This sample shouldn't contain nitrogen but I want to confirm it. Elements for analysis are C, H, and N.

Atlantic Microlab, Inc.

Sample No. SD-2-083A (13.8 mg) EKHFFF NP

6180 Atlantic Blvd. Suite M
Norcross, GA 30071
www.atlanticmicrolab.com

Company/School BROOKLYN COLLEGE
Dept. CHEMISTRY
Address 2900 BEDFORD AVE, ROOM # 350 NE
City, State, Zip BROOKLYN, NY, 11210

Professor/Supervisor: ANETA MIESZAWSKA
PO# / CC# AM06172021

Name SYLWIA DRAGULSKA Date 06/17/2021
Phone (718) 951-5000

Element	Theory	Found	
C	47.18	45.38	45.50
H	4.74	4.94	4.96
N	0.196	0.25	0.21

Single ☐ Duplicate ☒
Elements Present: C, H, N, O
Analyze for: C, H, N
Hygroscopic ☐ Explosive ☐
M.P. ______ B.P. ______
To be dried: Yes ☐ No ☒
Temp. ______ Vac. ______ Time ______
Rush Service ☐ Rush service guarantees analyses will be completed and results available by 5 PM EST on the day the sample is received by 11 AM.
Include Email Address or FAX # Below
sylwia.dragulska@brooklyn.cuny.edu

Date Received JUN 2 1 2021 Date Completed JUN 2 2 2021

Remarks: PHONE EXT. 18051
both samples contain the polymer so the theoretical calculation is the only estimation

Figure S4. Elemental analysis reports for (top) PLGA NPs and (bottom) EKHFFF NPs performed by Atlantic Microlab.

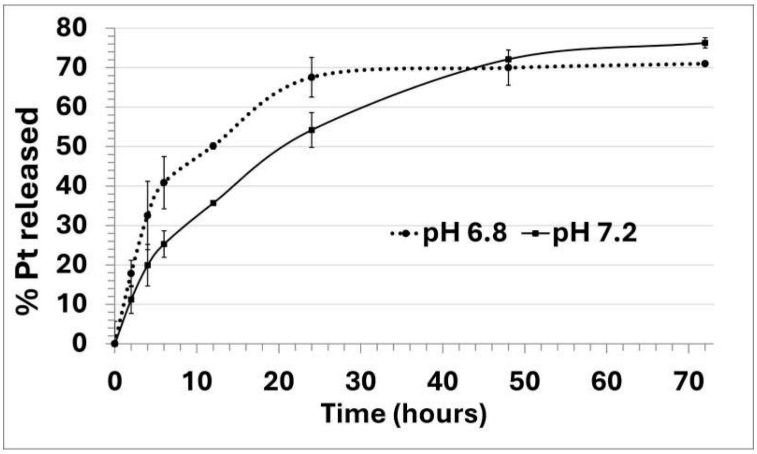

Figure S5. pH-dependent Pt(II) release profile of EKHFFF-Pt nanoparticles over 72 h at 37 °C. Data are presented as mean ±SD from three independent experiments (n = 3).

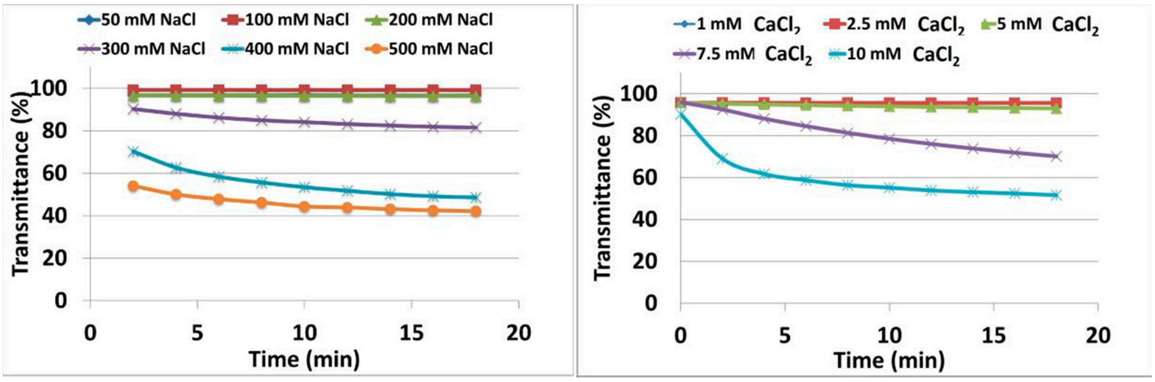

Figure S6. Transmittance kinetics of EKHFFF NP as a function of time at different ionic strengths of NaCl (left) and $CaCl_2$ (right). Data are presented as mean ±SD from three independent experiments (n = 3).

OvCA CLs	A2780	CP70	TOV-21G	SKOV-3	OVCAR-3	OV-90	ES-2
% EKHFFF-Cy5.5 NP	10.9 ± 9.54	10.6 ± 7.42	21.3 ± 14.4	7.3 ± 4.1	8.75 ± 4.87	8.95 ± 4.87	38.8 ± 9.82

PDCLs	PT217	PT927	PT1264	PT1310	PT1315	PT1372	PT1442	PT1446
%EKHFFF-Cy5.5 NP	95.8 ± 4.5	99.8 ± 4.8	98 ± 5.0	99.1 ± 4.9	94.1 ± 4.8	96 ± 4.9	99.9 ± 4.9	99.9 ± 4.9

Figure S7. Cellular uptake data of EKHFFF-Cy5.5 nanoparticles in ovarian cancer cell lines (OvCA) and patient-derived ovarian cancer cell lines (PDCLs) after 15 min incubation, determined by flow cytometry. Data are presented as mean ± SD from three independent experiments (n = 3).

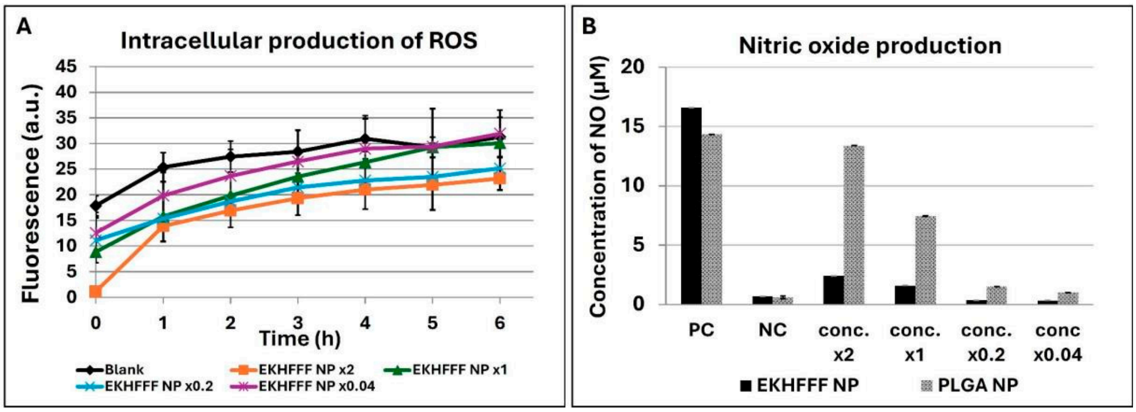

Figure S8. (A) Assessment of intracellular reactive oxygen species (ROS) production in RAW 264.7 cells following exposure to EKHFFF NP in different concentrations. (B) Nitrite accumulation in culture supernatants was quantified using the Griess reaction after 48 h exposure to nanoparticles. LPS (100 ng/mL) and PBS were used as positive and negative controls, respectively. Data are presented as mean ±SD from three independent experiments (*n* = 3).

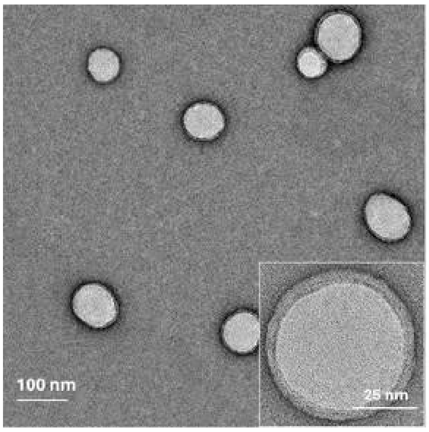

Figure S9. The TEM image for EKHFFF-Pt NP. The average nanoparticle size was 62.2 ±1.08 nm. Data are presented as mean ±SD from three independent experiments (*n* = 3).

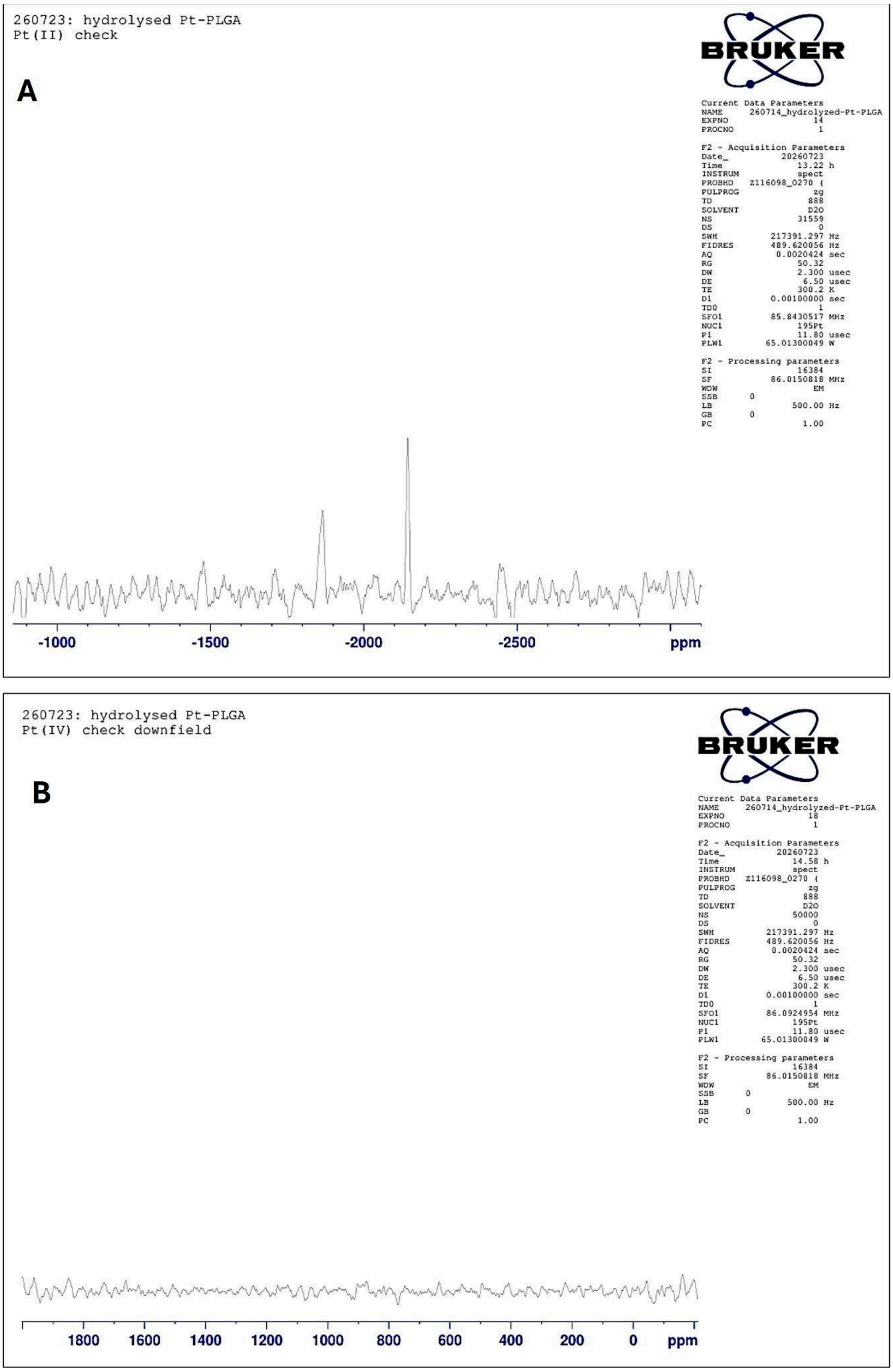

Figure S10. ^{195}Pt NMR spectra of the hydrolyzed PLGA–Pt(II) conjugate: **(A)** Pt(II) region showing the characteristic Pt(II) signal and **(B)** Pt(IV) region showing no detectable signal. Spectra were acquired on a Bruker Avance III HD spectrometer equipped with a 400 MHz UltraShield magnet and processed using TopSpin 3.5 software

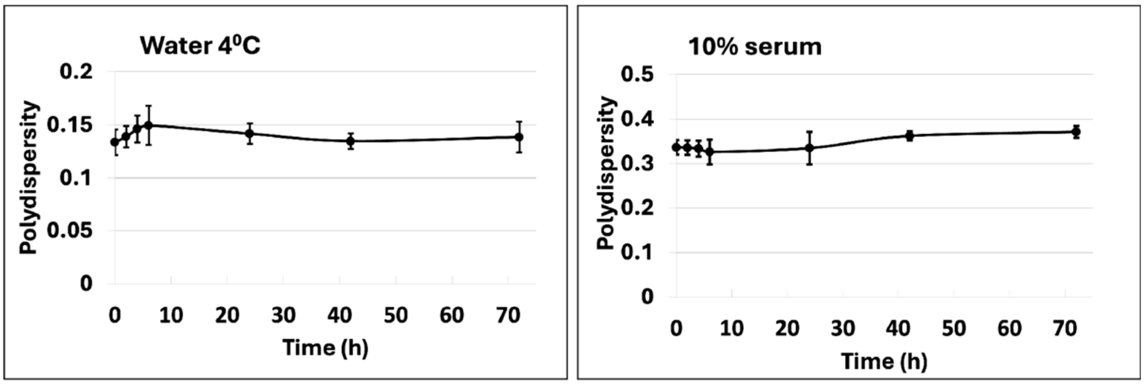

Figure S11. Polydispersity index (PDI) of EKHFFF NP under different conditions. Data are presented as mean ±SD from three independent experiments (n = 3).

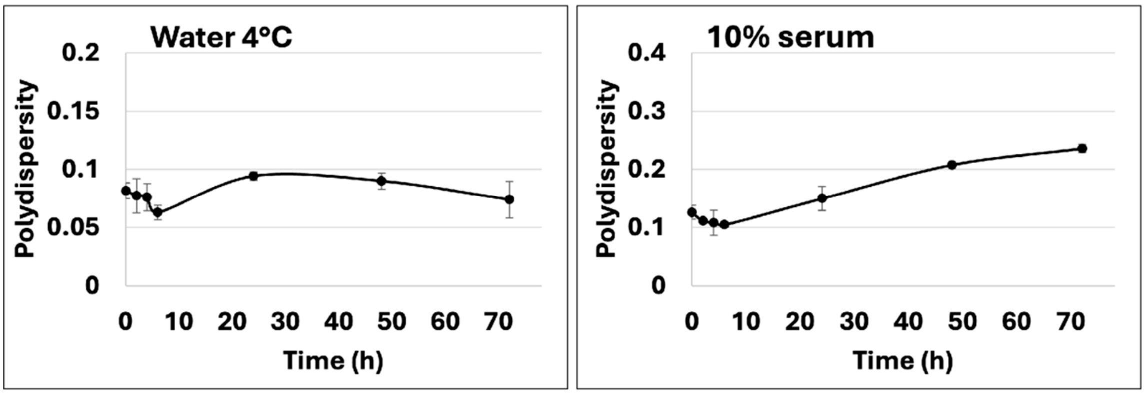

Figure S12. Polydispersity index (PDI) of EKHFFF-Pt NP under different conditions. Data are presented as mean ±SD from three independent experiments (n = 3).

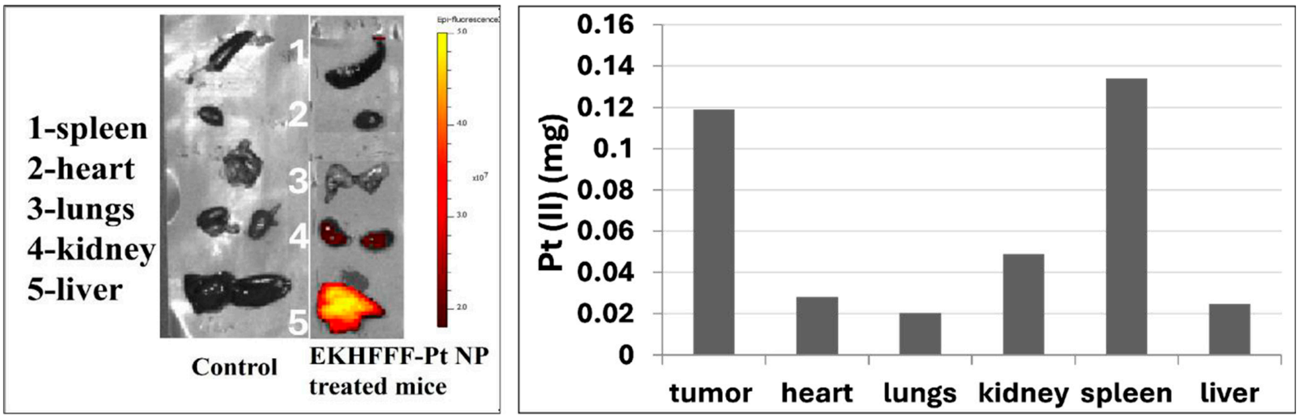

Figure S13. NIRF images of organs excised from mice after in vivo experiments (left) and corresponding Pt(II) mass (mg) in the organs mass (mg) (right).

Movie S1. Three-dimensional (3D) Imaris reconstruction of ES-2 cells treated with EKHFFF–Cy5.5 nanoparticles. The video is provided as a separate supplementary file.
